# Conservation of A-to-I RNA editing in bowhead whale and pig

**DOI:** 10.1371/journal.pone.0260081

**Published:** 2021-12-09

**Authors:** Knud Larsen, Mads Peter Heide-Jørgensen

**Affiliations:** 1 Department of Molecular Biology and Genetics, Aarhus University, Aarhus C, Denmark; 2 Greenland Institute of Natural Resources, Copenhagen K, Denmark; University of Toronto, CANADA

## Abstract

RNA editing is a post-transcriptional process in which nucleotide changes are introduced into an RNA sequence, many of which can contribute to proteomic sequence variation. The most common type of RNA editing, contributing to nearly 99% of all editing events in RNA, is A-to-I (adenosine-to-inosine) editing mediated by double-stranded RNA-specific adenosine deaminase (ADAR) enzymes. A-to-I editing at ‘recoding’ sites results in non-synonymous substitutions in protein-coding sequences. Here, we present studies of the conservation of A-to-I editing in selected mRNAs between pigs, bowhead whales, humans and two shark species. All examined mRNAs–NEIL1, COG3, GRIA2, FLNA, FLNB, IGFBP7, AZIN1, BLCAP, GLI1, SON, HTR2C and ADAR2 –showed conservation of A-to-I editing of recoding sites. In addition, novel editing sites were identified in NEIL1 and GLI1 in bowhead whales. The A-to-I editing site of human NEIL1 in position 242 was conserved in the bowhead and porcine homologues. A novel editing site was discovered in Tyr244. Differential editing was detected at the two adenosines in the NEIL1 242 codon in both pig and bowhead NEIL1 mRNAs in various tissues and organs. No conservation of editing of KCNB1 and EEF1A mRNAs was seen in bowhead whales. In silico analyses revealed conservation of five adenosines in ADAR2, some of which are subject to A-to-I editing in bowheads and pigs, and conservation of a regulatory sequence in GRIA2 mRNA that is responsible for recognition of the ADAR editing enzyme.

## Introduction

RNA editing is a molecular process in which nucleotide changes are introduced into an RNA sequence after transcription. Numerous editing events contribute to proteomic sequence variation [1, 2]. Tens of thousands of A-to-I sites were identified in mouse and millions in human [2–5]. Over 100 different types of RNA editing have been found and more than 340 functionally characterized proteins involved in RNA modification identified [6]. Most editing occurs within repetitive elements such as Alu elements and SINEs. RNA editing is found in tRNA, rRNA, mRNA, and miRNA molecules from both eukaryotes and prokaryotes [7]. RNA editing is widely distributed in the cell and occurs in the nucleus, and within mitochondria [8]. The most common type of RNA editing, contributing to nearly 99% of all editing events in RNA, is adenosine-to-inosine (A-to-I) editing by double-stranded RNA-specific adenosine deaminase (ADAR) enzymes [2].

In mammals, three different ADARs are found. The double-stranded RNA-specific adenosine deaminases ADAR1 and ADAR2 are both catalytically active, while ADAR3, a third member of the family, is catalytically inactive [1, 9–11]. ADAR1 and ADAR2 are ubiquitously expressed, with ADAR1 being highly expressed and responsible for most A-to-I editing activity [2]. The functional role of A-to-I RNA editing is believed to be dual: transcriptome diversifier and immune protector [2]. Using transcriptomic studies, a number of A-to-I editing recoding sites have been identified. The results are non-synonymous substitutions in numerous protein-coding sequences. RNA editing is highly evolutionary conserved, and some recoding sites are under positive selection and have functional and evolutionary importance. A-to-I editing levels differ both spatially and temporarily, and may vary across transcripts and tissues. Editing degrees can also be different throughout organism development. Editing levels can vary from 1 to 100% at any given site [5, 12].

Recoding and non-coding editing events are important for genome evolution and, when editing is deregulated, it may lead to disease. During translation the inosines created by editing are interpreted as guanosines instead of the adenosines encoded in the genome [13]. Editing is classified as ‘recoding’ if these adenosine to guanosine changes occur in protein-coding sequences and lead to non-synonymous substitutions that generate novel protein variants. Recoding is most common in neural tissues, and recoded sites are highly represented in transcripts that encode proteins with functions in the nervous system, exemplified by ion channels and neuroreceptors [14–16]. Among recoded transcripts with a function in neural tissues are GRIA2 and HTR2C.

GRIA2 (also named GluA2) is a glutamate receptor and a member of the family of glutamate receptors that are sensitive to alpha-amino-3-hydroxy-5-methyl-4-isoxazole propionate (AMPA). GRIA2 is the predominant excitatory neurotransmitter receptor in the mammalian brain, and it is activated in a variety of normal neurophysiologic processes. GRIA2 functions as part of a ligand-activated cation channel. These channels are assembled from four related subunits, GRIA1–GRIA4. The pre-mRNA encoding the GRIA2 subunit is subject to RNA editing, by ADAR2, within the second transmembrane domain, and this editing changes a glutamine to an arginine (Q607R) within the ion pore [17, 18]. The Q/R editing is crucial for normal AMPA receptor, and editing results in AMPARs almost completely Ca^2+^-impermeable. All GRIA2 subunits in the adult brain are Q/R-edited. GRIA2 pre-mRNA editing is essential for brain function in humans and animals examined, and defective GRIA2 RNA editing at the Q/R site leads to seizures. Lack of GRIA2 RNA editing is lethal in mice and impaired GRIA2 editing may associated with amyotrophic lateral sclerosis (ALS) aetiology [19]. Another edited GRIA2 site within the ligand-binding domain, an R/G mediated by ADAR1, is never completely edited [20].

The HRT2C transcript encoding the serotonin 2c receptor is also A-to-I edited. This editing affects the amino-acid composition of the second intracellular loop of the 5-HT_2C_R receptor, and the recoding leads to conformational changes that all decrease G-protein-coupling activity, agonist affinity and thus serotonin signalling [21–24]. Five adenosines in the HTR2C pre-mRNA, contained within 20 bases located on exon V are subjected to editing by ADAR enzymes. These adenosines are termed A to E sites. Editing at the A, B and C sites is mediated by ADAR1, whereas editing at the D site is catalysed by ADAR2 [25]. The E site (also named C′) is edited by both ADAR1 and ADAR2 enzymes. Theoretically, the A-to-I RNA editing of HTR2C pre-mRNA can generate up to 24 different receptor isoforms. The various isoforms each have specific activity. RNA editing also contributes to variations in splicing and intracellular trafficking of the 5-HT_2C_R receptor, thereby affecting receptor function [14, 26, 27].

Recoding also occurs in non-neural transcripts, such as the mammalian FLNA [7], AZIN1 [28] and NEIL1 [28] pre-mRNAs. Recoding of NEIL1, a DNA repair enzyme, results in a 30-fold reduction in the rate at which it removes thymine glycol from duplex DNA [29]. A-to-I editing has been investigated in pigs, and both global analyses and transcript-specific studies have been reported [30–34].

Alterations in A-to-I editing, hyperediting or hypoediting, of transcripts for oncogenes, tumor suppressor genes and their regulators might be a driver of cancer progression or/and simply a passenger alteration. For example, hyperediting of FLNB transcripts is seen in esophageal squamous cell carcinoma [35]. Several individual A-to-I editing events in different gene transcripts play critical roles in tumor formation [36]. Dysregulated RNA editing has been found in different types of cancers, and investigators often use it as a molecular marker [37–39].

Whales are the longest-living species of mammals and they have evolved mechanisms against diseases such as cancer [40]. Several evolutionary strategies for the development of cancer resistance have been proposed [41], including DNA damage repair, cellular senescence and specific adaptations to the environment [42, 43]. Improved immune systems, DNA repair mechanisms and other biological processes possibly favors cancer resistance and greater longevity in whales [44]. Positive selection and gene duplication in tumor suppressor genes might have promoted evolution of anti-cancer resistance, gigantism and longevity of whales [40].

The objective of the presented study was to investigate the conservation of A-to-I RNA editing in a selection of pre-mRNAs in pigs and bowhead whales, compared to editing of their human counterparts. The pig was chosen because of its evolutionary close connection with whales and as a terrestric living animal versus an aquatic. In particular, we studied recoding events in mRNA encoding in gene products involved in neural function and gene products associated with cancer development. The bowhead whale (*Balaena mysticetus*) is the longest-living mammal on earth and displays cancer resistance [45]. It is therefore of interest to investigate how A-to-I editing is in this organism differs from that in humans, and if it possibly contributes to cancer resistance.

Recoding of transcripts by A-to-I editing can give rise to protein variants in a similar way as missense mutations and thereby create a higher proteomic diversity. Some of these recoding events might represent a positive selection in genes associated with cancer resistance.

## Materials and methods

### Ethics

Housing of pigs and approval of experimental procedures was described by Henriksen et al. [46]. The pigs were sacrificed using an intravenous injection with 30 mg/kg pentobarbital.

The bowhead samples used in this project was collected by Mads Peter Heide-Jørgensen from The Greenland Institute of Natural Resources and University of Copenhagen and used under the CITES permission number 12GL1003387.

### Biological material

The bowhead whales sampled in this study were taken under the aboriginal subsistence whaling quota regulations implemented by the International Whaling Commission (IWC). The IWC ensure that hunted whale populations are maintained at healthy levels, and enable native people to hunt whales at levels that are appropriate to cultural and nutritional requirements in the long term. These principles encompasses the obligations for hunters and the competent authorities to conduct the hunt in a sustainable way and minimizing animal suffering associated with the hunting and killing methods. The bowhead whales were captured by local hunters in the Disko Bay using a harpoon gun and harpoons with an explosive grenade (Kongsberg, Norway). The sacrificed whales were transported to nearby harbours and dissected as described [47]. Bowhead whale organ and tissue samples were from four individuals: #322, #323, #501 and #502. Characteristics of the bowhead individuals such as estimated age, gender, size and place of capture are described by Heide-Jørgensen et al. [47].

Pig organ and tissue samples were dissected from three one-year old Danish Landrace pigs: #147, #3713 and #5621.

### Isolation of nucleic acids

Tissues from adult porcine organs and bowhead tissues were dissected and pulverized in liquid nitrogen after removal. Total RNA was isolated using the RNeasy method (Qiagen). DNA was isolated from biological samples according to standard purification protocols [48]. Synthesis of the cDNA used for cloning was performed as described in Henriksen et al. [49]. cDNAs used for expression analysis were synthesized from RNA isolated from various sources using random hexamer primers (Roche) and the manufacturer’s protocol. The integrity of the RNA samples was verified by ethidium bromide staining of the ribosomal RNA on 1% agarose gels. The quality of the DNA isolated from pig and bowhead tissues was examined by agarose gel electrophoresis.

### PCR amplification of NEIL sequences from pigs and bowhead whales

NEIL1-specific oligonucleotide primers derived from DNA sequences in exon 6 of the porcine and bowhead NEIL1 genes were used in a polymerase chain reaction (PCR). The sequences of the primers SSNEIL1-AIF, SSNEIL1-AIR, BMNEIL1-EF1, BMNEIL1-ER1, SANEIL1-AIF and SANEIL1-AIR are listed in S1 Table. PCR was performed with Invitrogen Platinum SuperFi Green DNA polymerase and 0.5-μM primers. We used the following PCR program to amplify porcine, bowhead and shark amplicons of 318 bp, 238 bp and 322 bp, respectively: 98°C for 30 secs followed by 32 cycles of 98°C for 10 secs and 72°C for 15 secs, finalizing with 72°C for 15 secs.

### PCR amplification of edited sequences for COG3, GRIA2, FLNA, FLNB and IGFBP7 mRNAs

The cDNA fragments containing the putative editing sites of COG3, GRIA2, FLNA and FLNB were amplified using the primers SSCOG3-AIF and SSCOG3-AIR, BMCOG3-AIF and BMCOG3-AIR, SSGRIA2-AIF and SSGRIA-AIR, BMFLNA-AIF and BMFLNA-AIR and BMFLNB-AIF and BMFLNB-AIR, and BMIGFBP7-AIF and BMIGFBP7-AIR. Primers SAGRIA2-F and SAGRIA2-R were used to amplify the shark GRIA2 sequences. See S1 Table for sequence information. PCR was performed with Invitrogen Platinum SuperFi Green DNA polymerase and 0.5-μM primers. We used the following PCR program to amplify porcine and bowhead COG3 and FLNA amplicons: a three-step protocol consisting of 98°C for 30 secs followed by 35 cycles of 98°C for 10 secs, annealing at 65–72°C for 20 secs and 72°C for 15 secs, and finalizing with 72°C for 15 secs. Annealing temperatures were 66°C for COG3, 54°C for GRIA2, and 72°C for FLNA and FLNB, respectively.

### PCR amplification of edited sequences for ADAR2, AZIN1, BLCAP, GLI1, SON and HTR2C mRNAs from pigs and bowheads

Reverse transcriptase–polymerase chain reaction (RT-PCR) was performed to test for the presence of A-to-I editing in AZIN1, BLCAP, GLI1, SON and HTR2C mRNAs. To amplify AZIN1 and BLCAP sequences from bowhead and pig, we designed primers, which could be used for both species. Primer sequences for BMAZIN-EDF, BMAZIN-EDR, BMBLCAP-EDF and BMBLCAP-EDR and amplicon sizes are listed in S1 Table. For GLI1, primers BMGLI1-EDF and BMGLI1-EDR were used; for SON, the primers SSSON-AIF and SSSON-AIR were used; and for HTR2C, we employed the primers BMHTR2C-AIF and HTR2C-AIR (S1 Table). PCR was performed with Invitrogen Platinum SuperFi Green DNA polymerase and 0.5μM primers. We used the following PCR program for BLCAP: 98°C for 30 secs followed by 35 cycles of 98°C for 10 secs and 72°C for 15 secs, finalizing with 72°C for 15 secs.

For AZIN PCR we used a three-step protocol consisting of 98°C for 30 secs followed by 35 cycles of 98°C for 10 secs, annealing at 65°C for 20 secs and 72°C for 15 secs, and finalizing with 72°C for 15 secs.

### PCR amplification of porcine huntingtin protein (HTT) 3’ untranslated region (UTR) edited sequences

We used three oligonucleotide primer sets specific to huntingtin protein (HTT) to examine five edited adenosines located within the porcine 3’ untranslated region (UTR) sequence: HTT-F1 and HTT-R1, HTT-F3 and HTT-R3, and HTT10711-F and HTT10711-R (S1 Table). PCR was performed with Invitrogen Platinum SuperFi Green DNA polymerase and 0.5-μM primers. We used the following PCR program to amplify the porcine HTT amplicons of 300 bp, 338 bp and 1054 bp: 98°C for 30 secs followed by 32 cycles of 98°C for 10 secs and 72°C for 15 secs, finalizing with 72°C for 15 secs.

### Estimation of A-to-I editing degree

The A-to-I editing degree was estimated by measuring top heights of the adenosine and guanine signals in the sequencing electropherograms. The editing degree was calculated as [G (mm)/(A (mm) + G (mm))] x 100.

### Expression analysis

Expression was determined by quantitative RT-PCR analysis. Organ and tissue samples for the spatial expression analysis were collected from three one-year old Danish Landrace pigs weighing 125–150 kg and three Landrace pigs aged 7, 11 and 12 years. Based on earlier observations [50], we used GAPDH as a reference gene in determining mRNA expression. For the quantitative real-time RT-PCR analysis of porcine and bowhead mRNA expression for the different transcripts, we isolated total RNA with MirVana miRNA Isolation Kit. Single-stranded cDNA was synthesized from 2.5 μg total RNA using oligo-dT primers and Superscript III reverse transcriptase, and the product was diluted 1:19 with water.

To the extent possible, we designed the primers to span the in-frame deletions, selecting probes from the EXIQON Human Probe Library. The primers and probes are listed in S1 Table. For a 10-μL PCR reaction, 3 μL cDNA template was mixed with 1x TaqMan Universal PCR Master Mix (Applied Biosystems), 300 nM forward and reverse primer and 125 nM designed probe/100 nM EXIQON Human Probe Library probe. We performed expression analysis on an ABI Prism 7900HT sequence detection system (Applied Biosystems), monitoring the increase in fluorescence caused by release of the probe reporter dye during amplification of the target sequence. The PCR reaction was run at default settings with initial denaturation and polymerase activation at 50°C for 2 min and 95°C for 10 min followed by 40–60 cycles at 95°C for 15 sec and 60°C for 1 min. The cDNA samples were run in technical triplicates. After termination of the reaction, the PCR product was verified by agarose gel-electrophoresis and sequencing.

We analysed expression data using the analysis of variance (ANOVA) procedure of the Statistical Analysis Software (version 8.2; SAS Institute Inc. Cary, NC). The equality of expression levels for selected genes between different tissues was tested for statistical significance using the Relative Expression Software Tool (REST) [50]. A probability level of *P* < 0.03 was set as statistically significant, and 50,000 randomization steps were implemented in each comparison [50].

### Bioinformatic analyses

The ORF Finder (http://www.ncbi.nlm.nih.gov/gorf/orfig.cgi) was used to identify open reading frames. Sequence analysis was performed using software online at NCBI (http://ncbi.nlm.nih.gov) and Expasy (http://expasy.org). The putative amino acid sequences were deduced using the Expasy translate tool (http://expasy.org/translate/). Homologues of CBLNs were retrieved from NCBI using blastx. ClustalW (http://www.genome.jp/tools/clustalw/) was used for sequence alignment. The theoretical isoelectric point (pI) and molecular weight (Mw) for CBLNs were estimated using Compute pI/Mw software (http://web.expasy.org/compute_pi/).

The sorting intolerant from tolerant (SIFT) algorithm was used to predict the potential impact of amino acid substitutions on the protein function of the gene products identified in this study (https://sift.bii.a-star.edu.sg/www/SIFT_seq_submit2.html). RegRNA 2.0 was used to identify functional RNA motifs in the transcripts included in this study (http://regrna2.mbc.nctu.edu.tw/). Putative target sequences for microRNAs in the *HTT* 3′UTR were identified by Target Scan Human 6.2 (http://targetsacn.org) and DIANA-microT-cds v5.0 (http://www.microrna.gr/microT-CDS.). The Mfold program was used as a tool for predicting the secondary structure of RNA (http://unafold.rna.albany.edu/?q=mfold/RNA-Folding-Form).

## Results and discussion

### NEIL1 editing in pigs and bowhead whales

**Fig 1:**
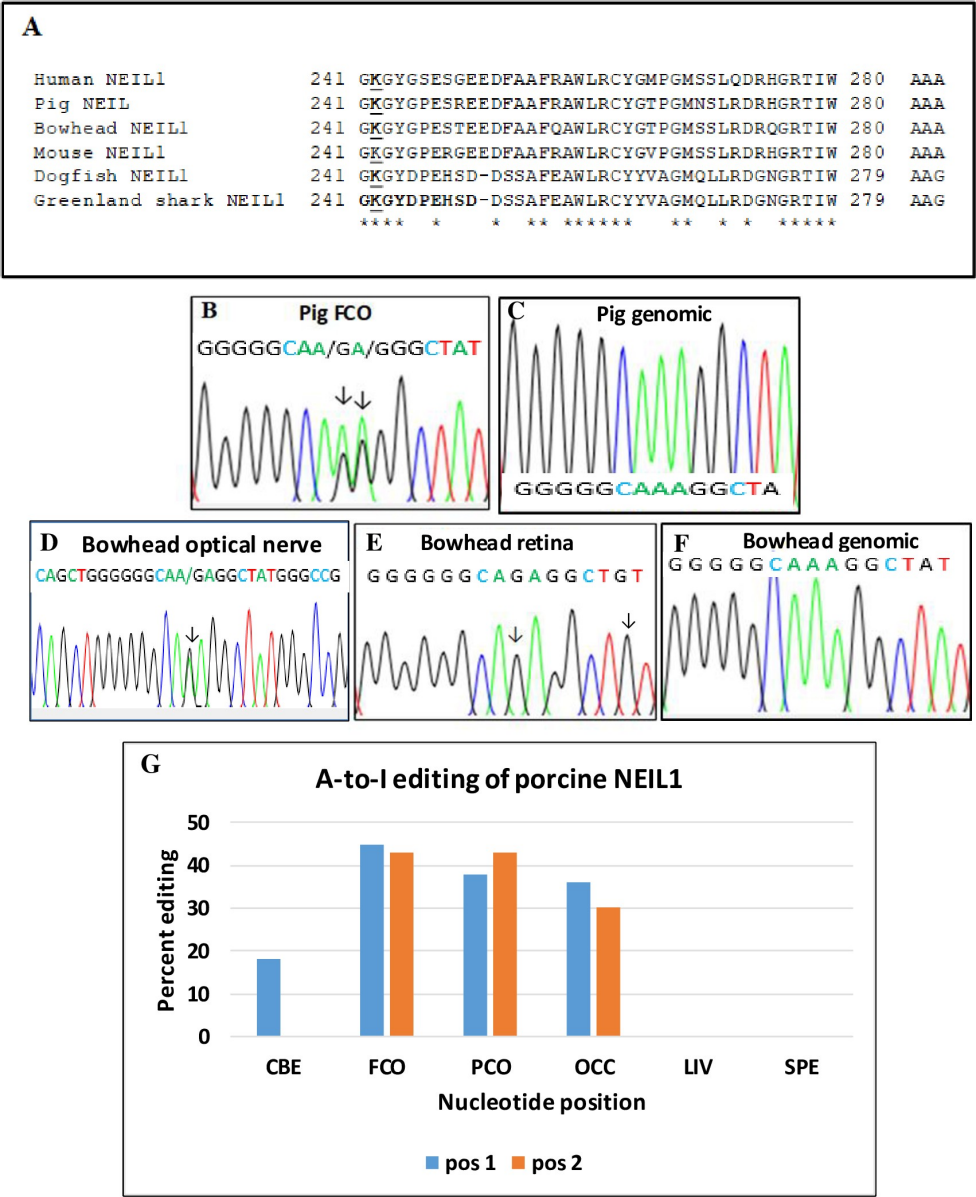
A) NEIL1 exon 6 alignment. Alignment of partial amino acid sequences for NEIL1 from pig, human, bowhead whale, mouse, spiny dogfish and Greenland shark. The following sequences were used in the alignment: Pig NEIL1, GenBank Accession No. NM_001163801; human NEIL1, GenBank Accession No. NM_001553; and mouse NEIL1, GenBank Accession No. NM_001159518. Sequence alignment was performed using the ClustalW program (http://www.genome.jp/tools/clustalw/). The numbers represent the positions of the amino acids in the respective protein sequences. Identical amino acids are indicated by asterisks. The edited amino acid position K242 in exon 6 is marked with bold and underlined letters. The codon sequence for K242 in NEIL1 of the five species is shown at the right. B–D) A-to-I editing of the porcine and bowhead NEIL1 pre-mRNA. Electropherograms showing a partial exon 6 nucleotide sequence of porcine NEIL1 cDNA with two adenosines subjected to A-to-I editing. The edited adenosines are indicated by arrows. E) Editing levels of the amino acid K242 position of the porcine NEIL1 pre-mRNA from various tissues and organs. F) Partial sequence of the bowhead NEIL1 mRNA. G) Histogram showing the quantification of A-to-I editing of adenosines in the K242 codon in porcine NEIL1 mRNA. Abbreviations used: CBE, cerebellum; FCO, frontal cortex; PCO, parietal cortex; OCC, occipital cortex; LIV, liver; SPE, spleen.

Using RT-PCR and RNA purified from organs and tissues of pigs, bowhead whales, spiny dogfish (*Squalus acanthias*) and Greenland sharks (*Somniosus microcephalus*), we amplified short NEIL1 cDNA fragments containing the recoding site. An alignment of the different amplicons shows a very high amino acid homology between the mammalian NEIL1 sequences and significantly lower homology between the mammalian and the two shark sequences from spiny dogfish and Greenland sharks (Fig 1A). The nucleotide triplet encoding the conserved lysine 242 residue is AAA in the mammalian sequences and AAG in the shark sequences (Fig 1A). The results from Sanger sequencing of the NEIL1 amplicons revealed conservation of A-to-I editing in K242 of the second and third adenosine in pig NEIL1 compared with the human NEIL1 transcript (Fig 1B and 1C and Table 1) and conservation of editing of the second adenosine in the bowhead NEIL1 pre-mRNA (Fig 1D–1F). An examination of six different porcine tissues revealed editing in both adenosines (position 2 and position 3) in the NEIL1 K242 codon in cortices, while only editing of the adenosine in position 2 was found in cerebellum (Fig 1G). In contrast, no editing of NEIL1 mRNA was detected in liver (LIV) and spleen (SPE). In the bowhead retina, an editing degree of 100% was seen, while the editing in the bowhead optical nerve was around 50% (Fig 1D and 1E). A potential editing of shark NEIL1 pre-mRNA was examined in spiny dogfish liver, brain, kidney and ovary and Greenland shark spinal cord and brain. No A-to-I editing of NEIL1 mRNA was observed in the either of the two shark species. An analysis of A-to-I editing of NEIL1 mRNA in four different pig brain tissue samples and pig liver and spleen samples showed editing values between 18% and 45% in the cerebellum and the occipital, parietal and frontal cortices for the adenosine in the second position in the AAA codon (Fig 1G). For the adenosine in the third position of the triplet, editing was seen in the cortices (30–42%), but not in the cerebellum. No NEIL1 editing was detected in pig liver, spleen or sperm cells. A novel editing site was discovered in the Tyr244 (TGT) residue of the bowhead NEIL1 mRNA (Fig 1E and 1F).

**Table 1:** Conservation of A-to-I recoding editing sites in bowhead, pig and human. AA, amino acid substitutions.

Gene symbol	AA	Bowhead	Pig	Human
NEIL1	K/R	+	+	+
COG3	I/V	+	+	+
GRIA2	Q/R	+	+	+
FLNA	Q/R	+	NA*	+
AZIN1	S/G	+	NA	+
BLCAP	Y/C	+	NA*	+
GLI1	K/R	+	+	+
SON	R/G	+	+	+
HTR2C	Y/C, Q/R, K/R	+	+**	+
IGFBP7	K/R, R/G	+	+***	+
FLNB	Q/R	+	NA	+
EEF1A	T/A, I/V	-	NA	+
KCNB1	I/V	-	NA	+

AA, amino acid substitution; NA, not analyzed in this study

* Editing demonstrated in Refs. [30, 82]

** Ref. [33]

** Ref. [32]

The editing of NEIL1 was identified by a transcriptome sequence analysis from various human tissues [28]. The predicted recoding in the pre-mRNA for the DNA repair enzyme NEIL1 (lysine 242 AAA codon edited to AIA codon for arginine) was confirmed by Yeo et al. [29]. NEIL1 is involved in base excision repair of oxidized base lesions; it catalyses the cleavage of the N-glycosidic linkage to the 2’-deoxyribose [51]. The recoding of NEIL1 pre-mRNA causes a lysine to arginine change in the lesion recognition loop of the protein. The editing has a functional consequence, and unedited and edited forms of NEIL1 have distinct enzymatic properties [29]. The edited form removes thymine glycol from duplex at a slower rate (DNA 30 times more slowly) than the form encoded in the genome, whereas editing enhances repair of the guanidinohydantoin lesion by NEIL1 [29]. The editing of the NEIL1 recoding site, located in exon 6, is predominantly performed by ADAR1, and editing at this position is increased in human cells treated with interferon α [29]. In multiple myeloma, many transcripts are aberrantly hyper-edited because of the overexpression of ADAR1, and the recoded NEIL1 protein shows defective oxidative damage repair capacity and loss-of-function properties [52]. These data revealed novel insights into molecular pathogenesis at the global RNA level. The NEIL1 editing is also found in chickens and sheep [53, 54]. The target duplex secondary structure and A–C mismatch at the recoding site is conserved in other vertebrate NEIL1 mRNAs, including from mouse, horse and dog, suggesting NEIL1 pre-mRNA editing also occurs in these species. In relation to this, it is interesting that the two elasmobranch species included in this study do not show any conservation of NEIL1 mRNA. The mammalian species have, via the A-to-I editing, evolved at least two isoforms of the NEIL1 enzyme, some of which are beneficial for specific conditions in DNA repair.

### COG3 mRNA editing

**Fig 2:**
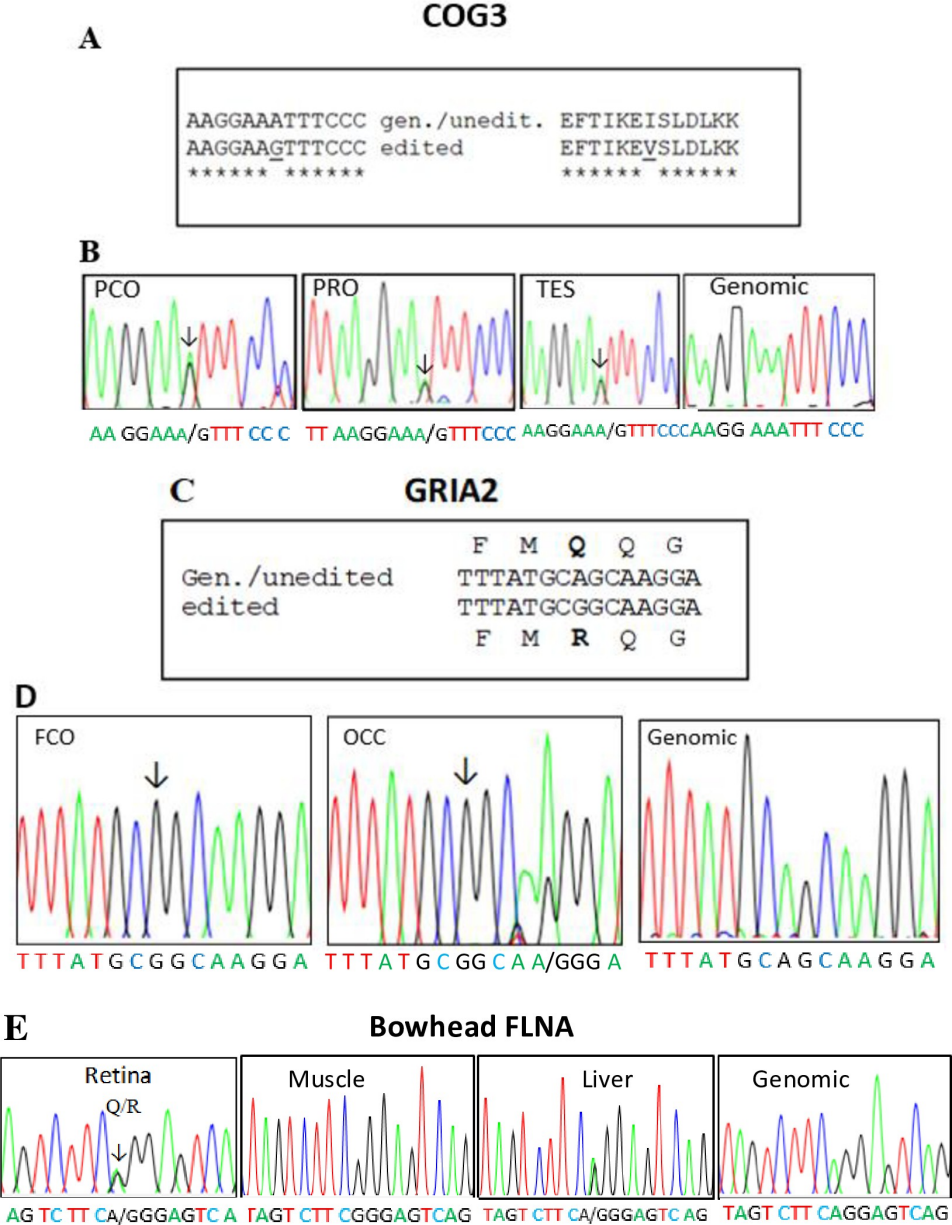
A) Porcine COG3 nucleotide alignment showing the genomic/unedited sequence (upper line) and the edited sequence below. B) Electropherograms showing editing in porcine parietal cortex (PCO), prostate (PRO) and testis (TES). The adenosine subject to editing is indicated by an arrow. Editing degrees are shown below. C) Porcine GRIA2 nucleotide alignment displaying the genomic/unedited sequence (upper line) and the edited sequence below. D) Electropherograms showing the porcine GRIA2 sequences with editing in frontal cortex (FCO) and occipital cortex (OCC). E) Electropherograms showing editing of bowhead FLNA pre-mRNA in retina and optical nerve.

Using RT-PCR cloning we examined A-to-I mRNA editing of the COG3 transcript in the pig. The editing site in the porcine COG3 mRNA and the recoding of amino acid 635 are shown in Fig 2A. The editing of this particular adenosine in the ATT codon was shown to be conserved between pig and bowhead (S1 Fig) and human. The recoding within this codon results in an amino acid substitution from an isoleucine to a valine residue (I635V). The degree of editing was determined in three different porcine tissues: parietal cortex, prostate and testis (Fig 2B). Interestingly, we found COG3 pre-mRNA editing in all tissues with similar values: parietal cortex (40%), prostate (47%) and testis (47%). These values are similar to those reported from analysis of testicular editing in mice [55]. Snyder et al. [55] performed a computational analysis of whole testis and cell-type specific RNA-sequencing data followed by molecular conformation. They showed that A-to-I RNA editing occurs in both the germline cells and in somatic Sertoli cells in the COG3 pre-mRNA. In addition, they demonstrated that global ADARB1 knockout mice have a complete loss of A-to-I editing in spite of normal germ cell development. In conclusion, ADARB1 mediates A-to-I RNA editing in the testis, and these editing events are dispensable for male fertility in inbred mice.

The A-to-I RNA editing of the COG3 mRNA was also analysed in mRNA from various bowhead whale tissues. Editing in the I635 codon was found in all samples examined: muscle, liver, kidney, optical nerve and retina (S1 Fig and Table 1). Very high editing values of 80 to 100% were found in the adenosine in the first position of the codon in liver, kidney and optical nerve, while a lower value (33%) was detected in the retina (S1 Fig). No editing of the adenosine in position 1 was seen in muscle. In addition, editing was also found in the preceding E634 codon, with the adenosine in the third position edited in the liver only, with an editing degree of around 60% (S1 Fig). This editing is not recoding. The differential editing of COG3 editing observed in pig and bowhead suggests that differences in editing could have functional importance in various tissues. The COG3 gene encodes a component of the conserved oligomeric Golgi (COG) complex, which is composed of eight different subunits and is required for normal Golgi morphology and localization [56]. Defects in the COG complex lead to multiple deficiencies in protein glycosylation. The COG3 protein is involved in ER–Golgi transport. RNA sequencing of porcine hypothalamus and liver revealed A-to-I editing of the component of oligomeric Golgi complex 3, or COG3, transcript (ENSSSCG00000027815: g.4525A>G, ss1985401087). The results were validated through Sanger sequencing and by genotyping using the pyrosequencing method [57]. Similarly, RNA sequencing of liver and white adipose tissue from chickens showed the editing of COG3 mRNA was conserved. Interestingly, the edited position of the COG3 pre-mRNA displayed a recoding level that was significantly impacted by genetic background, age and feeding. Holmes et al. [58] also identified a conserved editing site in rat COG3. No change in COG3 pre-mRNA editing was observed with age in humans or in healthy rats. The recoding of COG3 pre-mRNA resulting in the I635V substitution has previously been described in humans, mice, rats and cows [59–62] and more recently in chickens [53, 62], proving that at least some RNA editing conservation occurs across vertebrate species. Bakhtiarizadeh et al. [61] reported COG3 editing in colon, heart, kidney, liver, lung, spleen and testes tissue but not in the brain or skeletal muscle. This is in contrast to our observation of COG3 editing in the pig frontal cortex. According to the SIFT prediction tool, the recoding of the component of COG3 –an Ile83Val change to a key molecule in protein metabolism–is predicted to be functional but tolerated. A sequence analysis performed by RegRNA2.0 revealed that the modification lies in an exon enhancer region motif, GGAAG, involved in the promotion of alternative splicing [63], which is likely linked to different biological functions conditional on tissue type.

### GRIA2 mRNA editing

We examined the porcine counterpart of the Q/R A-to-I editing site at codon 586 previously described for human GRIA2 pre-mRNA. Alignment of the genomic/unedited sequence and the edited sequence of the partial GRIA2 mRNA is shown in Fig 2C. RT-PCR cloning and sequencing revealed editing of GRIA2 and recoding of amino acid 586 (Q586R) encoded by the porcine GRIA2 mRNA (Table 1). Editing degrees of 100% were seen in in all brain tissues examined (Fig 2D). The editing was also conserved in bowhead brain samples (Table 1). We also examined a possible editing of GRIA2 mRNA in the spiny dogfish and Greenland shark. In brain tissue from the spiny dogfish and Greenland shark, we observed editing degrees of 33% and 20%, respectively (S2 Fig). Editing of GRIA2 mRNA was also seen in the spiny dogfish olfactory bulb (26%) and the Greenland shark spinal cord (10%), while no editing was found in the Greenland shark’s olfactory rosette.

The best characterized A-to-I editing site occurs in the GluA2 AMPA receptor subunit, also called GRIA2. The editing, which occurs in codon 586, changes a glutamine residue to an arginine [64]. The unedited form of GRIA2 receptor, with a glutamine residue, allows both sodium and calcium to permeate. Editing of GRIA2 at codon 586 results in an arginine which makes the receptor impermeable to calcium and also decreases its conductance [65–67]. Excessive calcium permeation through the GRIA2 receptor would be expected to have very negative effects on intracellular signalling. Editing at the GRIA2 Q/R site occurs at close to 100%. Elimination of GRIA2 editing at this site by ADAR2 knock-out is lethal in mice as they die shortly after birth [68]. All mammalian GRIA2 genes examined so far specify a Q (CAG) codon at the Q/R position 586. Our observations with editing of the bowhead GRIA2 mRNA demonstrates the conservation of this event. Our examination of the GRIA2 sequences from the spiny dogfish and Greenland shark revealed a CAG codon encoding a glutamine residue at the Q/R position in both species. However, we did not observe any editing in the examined organs and tissues (liver, muscle and spinal cord). Kung et al. [69] examined the GRIA2 sequence from teleost fish and spiny dogfish and found an R residue in hagfish and some teleost. In contrast, the spiny dogfish contains a glutamine residue. The lack of editing of the shark GRIA2 could be explained by the absence of A-to-I editing regulatory sequences. GRIA2 is a glutamate receptor and the predominant excitatory neurotransmitter receptor in the mammalian brain, and it is activated in a variety of normal neurophysiologic processes. The pre-mRNA encoding the GRIA2 subunit is subject to RNA editing, by ADAR2, within the second transmembrane domain, and this editing changes a glutamine to an arginine (Q607R) within the ion pore [17, 18]. Q/R editing renders AMPARs virtually Ca^2+^-impermeable, which is important for normal AMPA receptor function. Thus, all GluA2 subunits in the adult brain are Q/R-edited. Human and animal studies suggest that pre-mRNA editing is essential for brain function, and defective GRIA2 RNA editing at the Q/R site leads to seizures, is lethal in mice and may be relevant to ALS aetiology. Recoding of the GRIA2 Q/R site is essential for post-natal viability in mice [68]. It is therefore surprising that the degrees of editing observed in the shark brain are less than 100%.

### FLNA and FLNB mRNA editing

We observed that A-to-I editing of the CAG triplet in the FLNA mRNA that encodes the Q2341 residue in filamin A was conserved in the bowhead retina, muscle and liver whereas no or very little editing was seen in the kidney and optical nerve (Fig 2E). With subsequent analyses we also observed editing in muscle (100%) and liver (45%) but no editing in the bowhead kidney. The human FLNA mRNA encoding filamin A is edited at a single adenosine in exon 42, which results in a Q-to-R (Q2341R) substitution in the Ig-repeat of the encoded protein [7, 70]. This particular site is located in the interactive rod 2 domain, and therefore editing could affect the interaction between filamin A and other proteins. Editing of FLNA is associated with both cardiovascular disease and psoriasis [71–73]. The filamin A mRNA editing is not limited to the central nervous system but also occurs outside neuronal tissues [72].

The pre-mRNA encoding FLNB is subjected to A-to-I editing leading to a glutamine (Q) for arginine (R) exchange [28]. Using RT-PCR we observed conservation of FLNB editing in bowhead (Table 1 and S3 Fig). By the sequencing analysis we found varying editing of FLNB mRNA in muscle (26%), retina (15%), liver (37%, and kidney (0%). The editing values are similar to those demonstrated for mouse FLNB [74]. We did not find any editing of the S/G immediately upstream of the Q/R site as described in mouse [74].

### IGFBP7 mRNA is edited in bowhead

Sequencing of RT-PCR products with gene-specific primers for IGFBP7 revealed editing of the bowhead transcript from liver, retina and muscle (S4 Fig). Editing was observed in codons for R78 (AGG) and K95 (AAG), while no editing was seen in K97 (AAA). For position R78, the highest editing rate of 85% was found in muscle. For the second adenosine in K95, the highest editing values was 92% seen in muscle (S4 Fig). In an earlier study, we demonstrated editing of porcine IGFBP7 mRNA [32]. Hence, the editing of IGFBP7 seems to be conserved in mammals.

### KCNB1 and EEF1A transcripts are not edited in bowhead

Using RT-PCR amplification and sequencing of partial transcripts we examined the potential A-to-I editing of KCNB1 and EEF1A in bowhead. As shown in Table 1, no conservation of editing was seen in KCNB1 and EEF1A amplicons originating from bowhead liver, kidney, muscle, retina and optical nerve.

### AZIN1 is edited in bowheads

We cloned the full-length AZIN1 cDNA coding sequence from the bowhead liver using RT-PCR. We then aligned the deduced amino acid sequence of the bowhead AZIN1 with its human counterpart. The alignment showed a very high homology between the two mammalian AZIN1 proteins, with an amino acid identity of 95% (S5 Fig). Among the 20 amino acid differences, we identified codon 367 to be an asparagine residue in the human AZIN1 but a serine residue in the bowhead sequence. Hence, the asparagine could be a positive selection event to protect the bowhead from developing cancer. However, the asparagine codon (AAC) is subject to A-to-I editing at both adenosine positions, meaning that the asparagine residue can be replaced by a serine and a glycine. A closer look at the electropherogram of the bowhead AZIN1 sequence showed a G/_A_G/_A_C codon. This codon 367 is subject to A-to-I RNA editing. The bowhead AZIN1 genomic sequence displayed an AAC codon.

**Fig 3:**
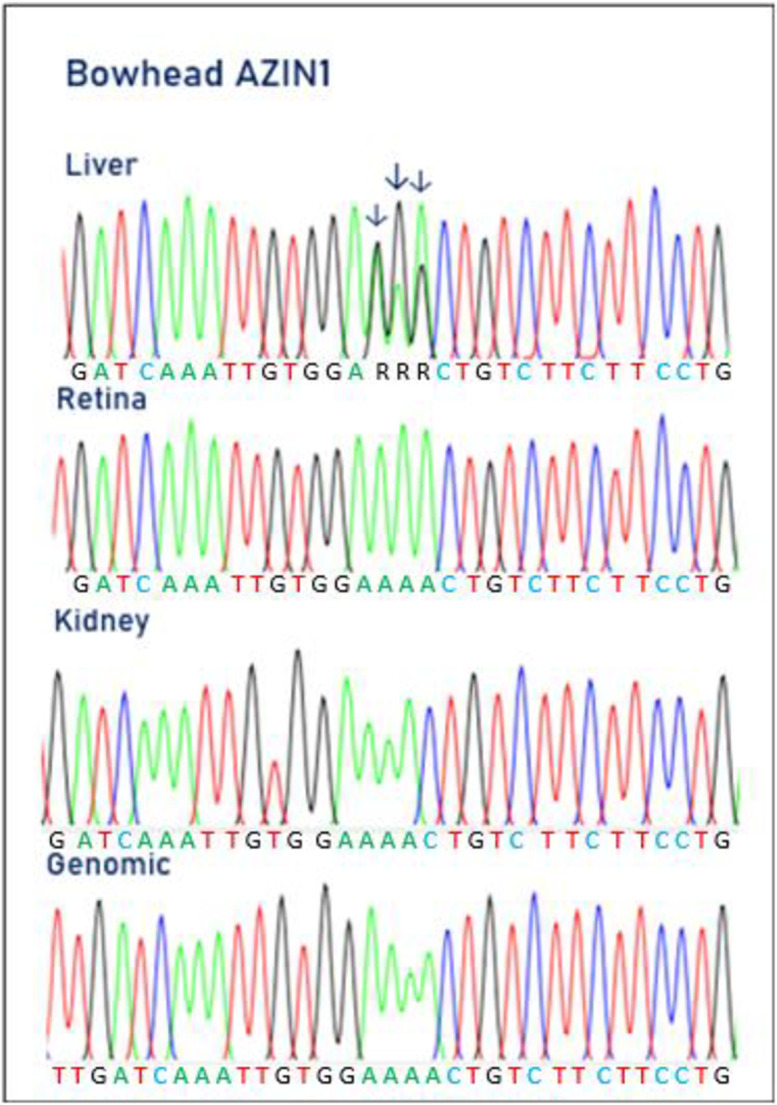
Electropherograms showing AZIN1 pre-mRNA editing in bowhead liver, retina and kidney. Electropherograms showing editing in liver (upper panel), retina (middle panel) and kidney (lower panel). The three adenosines subject to editing, within codon number 367, are indicated by arrows.

A semi-quantitative determination of the degree of editing degree of the bowhead AZIN1 mRNA in different tissues revealed editing in the liver with average values for the first adenosine of 75% and for the second adenosine of 48% (Fig 3 and Table 1). No editing was detected in AZIN1 transcripts from muscle, retina and kidney. We did not examine for bowhead AZIN1 editing in the heart or colon, and therefore we cannot report on the editing in these organs. In addition, we also saw A-to-I editing in the third position in codon 366 (GAG/_A_) of liver AZIN1 pre-mRNA. However, both the GAA and GAG encode the amino acid glutamic acid, so there is no change in the AZIN1 protein. An editing degree of around 55% was seen for this third position adenosine in codon 367 in liver AZIN1 pre-mRNA only. The consequences of A-to-I editing of the AZIN1 transcript, resulting in recoding, differ between bowhead whales and humans. Bakhtiarizadeh et al. [61] recently demonstrated conservation of AZIN1 editing in cows.

The antizyme inhibitor 1 (AZIN1) encoded by the AZIN1 gene regulates antizymes. Antizymes (AZ) are proteins, which promote degradation of ornithine decarboxylase (ODC), the key enzyme in the pathway of polyamine biosynthesis [75]. Antizymes are negative regulators of polyamine biosynthesis and transport into cells [76]. Antizymes are in turn regulated by AZIN1 and AZIN2 antizyme inhibitors. AZIN1 and AZIN2 are homologues of ODCs that bind to antizymes and counteract their negative effect on ODC [77]. AZIN1 is widely expressed in human organs and tissues and is involved in polyamine metabolism and cell growth [78]. AZIN1 interferes with the formation of the antizyme–ODC complex, thereby contributing to cancer cell development. Antizymes disrupt ODC homodimers, target ODC for degradation by the 26S proteasome and inhibit polyamine uptake. Overexpression of AZIN1 generates an increase in ODC activity, accelerating the formation of polyamine, triggering gastric and breast cancers and the development of hepatocellular carcinoma. A-to-I editing of AZIN1 pre-mRNA leads to a serine glycine substitution at residue 367 of AZIN1, which creates a conformational change. The edited AZIN1 binds to antizymes with a higher affinity than the wild-type AZIN1. This means that edited AZIN1 sequesters antizymes to block the degradation of ODC and cycline D1, thus promoting cell proliferation possibly leading to cancer onset. Dysregulation of RNA editing is associated with the development of different cancer types such as hepatocellular carcinoma, gastric cancer, and colorectal cancer [35, 79–81]. RNA editing of AZIN1 pre-mRNA frequently confers a gain-of-function phenotype through A-to-I conversions, which can promote ODC and polyamine accumulation–conditions that are associated with aggressive tumours.

### BLCAP editing in bowheads

**Fig 4:**
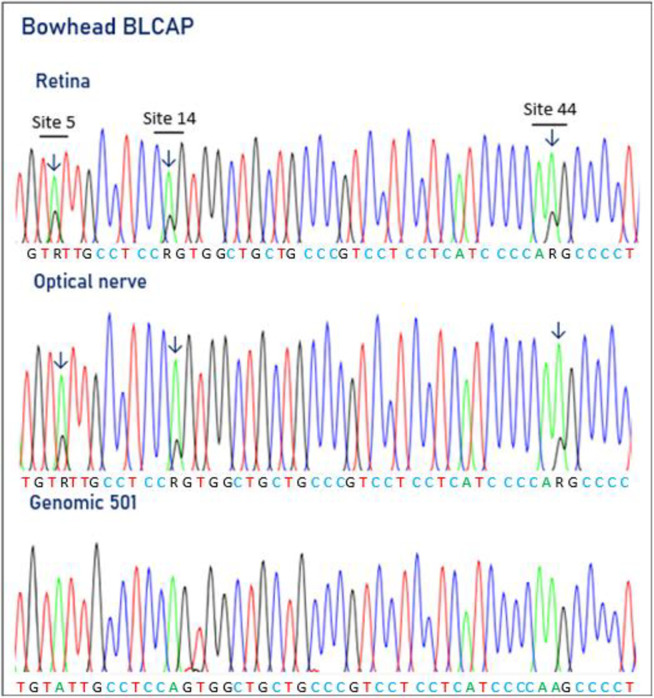
Electropherograms showing BLCAP pre-mRNA editing in bowhead retina and optical nerve. For comparison, the genomic sequence is shown below. Electropherograms showing editing in retina (upper panel) and optical nerve (middle panel). The adenosines subject to editing, in sites 5, 14 and 44, are indicated by arrows.

The potential A-to-I RNA editing of BLCAP mRNA, previously demonstrated in humans, was examined by RT-PCR on cDNA synthesized from different bowhead tissues. Editing of BLCAP mRNA was identified in the bowhead optical nerve and retina, with editing degrees at three adenosine positions of between 17% and 27%. Editing was observed at three adenosine positions, referred to as site 5, site 14 and site 44 (Fig 4 and Table 1). These adenosines are located in exon 2 of the BLCAP mRNA and are all recoded by editing and given names according to the amino acid change they produce: Y2C (site 5), Q5R (site 14) and K15R (site 44). The editing values for these sites were 24%, 20% and 27% in the optical nerve and 19%, 20% and 17% in the retina, respectively. No editing of BLCAP mRNA was seen in bowhead liver, kidney or muscle. The editing of BLCAP seems to be, at least for some recoding positions, conserved among mammals. Previously, BLCAP editing had been demonstrated in mice, pigs and sheep [7, 30, 53, 82, 83].

The BLCAP transcript, which is ubiquitously expressed, encodes the bladder cancer–associated protein (BLCAP), also named bladder cancer 10 kDa protein (BC10). Accumulating evidence suggests that BLCAP may function as a tumour suppressor in carcinogenesis mediated through regulation of cell proliferation and survival [84–87]. BLCAP is down-regulated in bladder cancer, in kidney cancer and in cervical carcinoma [84, 88, 89]. The BLCAP protein consists of 87 amino acids and has two transmembrane regions [89]. The BLCAP gene is highly conserved, and the coding nucleotide sequence of BLCAP is almost identical in the bowhead and the human BLCAP, with only four out of 264 nucleotides differing between the two. The human BLCAP is subjected A-to-I RNA editing at multiple adenosine sites [7, 89, 90]. The human BLCAP pre-mRNA contains three recoding editing sites in exon 2, 11 editing sites in intron 1, and three editing sites in the 5’UTR [90]. Differential editing degree was observed in various examined tissues. Chen at al. [79] also reported a general decrease in BLCAP editing in astrocytomas, bladder cancer and colorectal cancer compared with normal tissues. Aberrant BLCAP editing was also shown in other cancers such as tumour–specific editing patterns in lung cancer, oral cavity cancer, brain cancer and urinary bladder cancer [91, 92]. Hu et al. [93] reported over-editing in tumours of hepatocellular carcinoma. The over-editing of the BLCAP transcript very likely promotes cell proliferation, both *in vitro* and *in vivo*, by enhancing the phosphorylation of AKT, mTOR and MDM2 and inhibiting the phosphorylation of TP53. It is speculated that the A-to-I over-editing of BLCAP may serve as a driver of hepatocellular carcinoma through activation of the AKT/mTOR signal pathway [94]. Recently, Chen et al. [94] demonstrated a differential A-to-I editing of BLCAP adenosine positions site 5, site 14 and site 44 in the coding sequence of BLCAP in cervical cancer, in cancerous tissue and adjacent cervical tissues. The editing of these three sites were closely correlated. Two of the editing sites, 5 and 14, were mapped to the key YXXQ motif of BLCAP, which binds to the SH2 domain of signal transducer and activator of transcription 3 (STAT3). STAT3 is a transcription factor that regulates a variety of cellular activities. In its phosphorylated form, STAT3 migrates to the nucleus, where it recognizes STAT3-specific DNA binding elements. Among these are Bcl-2 family proteins, cyclins and matrix metalloproteinases, the last of which are associated with anti-apoptosis, pro-proliferation, induction of angiogenesis, promotion of metastasis and evasion of anti-tumour immunity [94]. Chen et al. [94] demonstrated that BLCAP interaction with STAT3 inhibits phosphorylation of STAT3, and that in cervical cancer cell lines A-to-I edited BLCAP lost its ability to inhibit STAT3 activation. The edited versions of BLCAP, CCLQ and CCLR, in contrast to the unedited YCLQ, partly or almost fully failed to inhibit STAT3 phosphorylation. It was concluded that A-to-I editing of BLCAP drives the anti-tumorigenic BLCAP to a loss-of-function, which might facilitate the initiation of cervical cancer. Given that BLCAP pre-mRNA editing was detected only in bowhead retina and optical nerve tissues, the absence of editing in other organs and tissues could have a protective role that contributes to cancer resistance in the bowhead. However, a much more detailed analysis of more organs and tissues is needed to confirm this.

### GLI1 editing in bowheads

**Fig 5:**
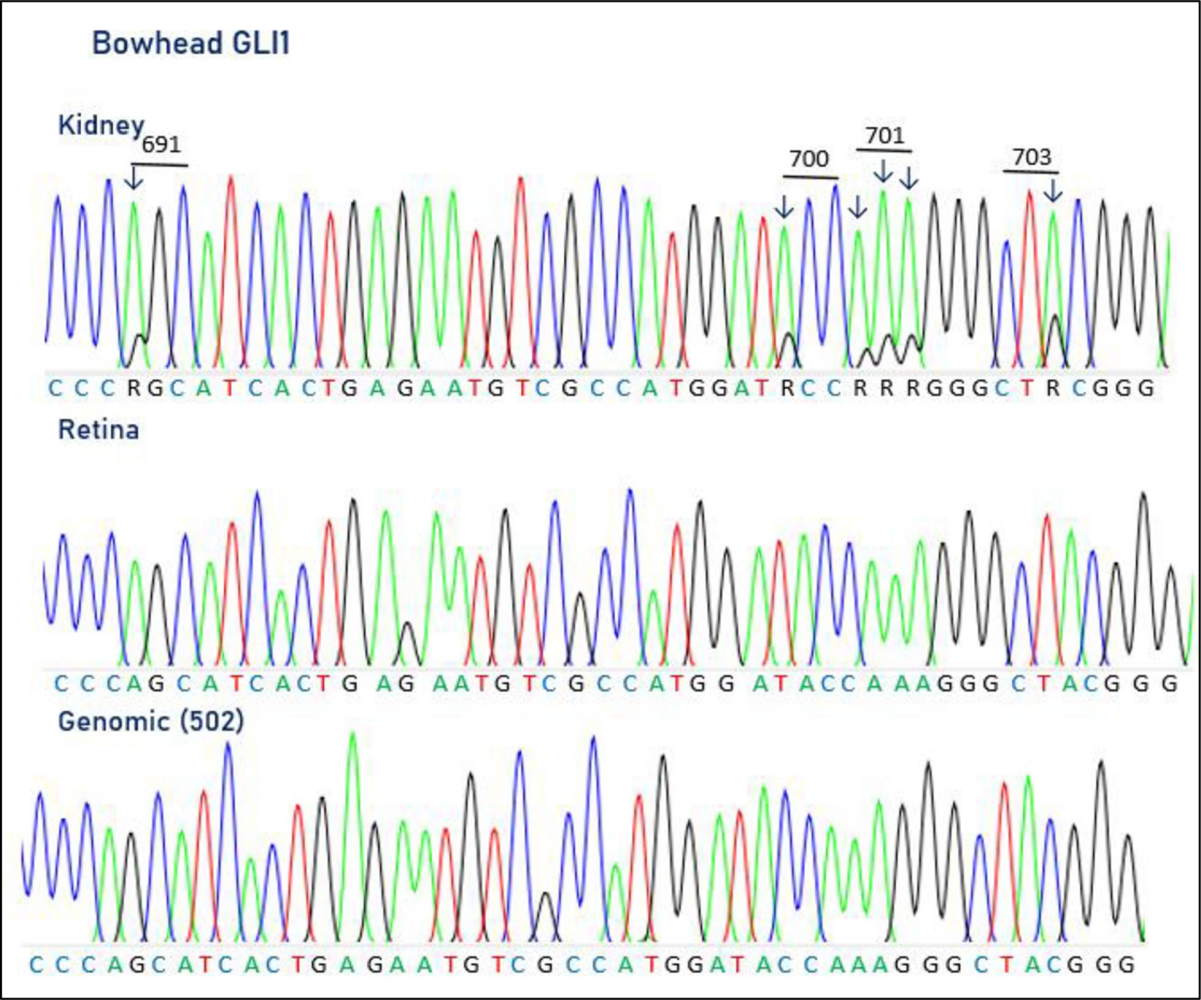
Electropherograms showing GLI1 pre-mRNA editing in bowhead kidney (upper panel) and retina (middle panel). For comparison, the genomic sequence is shown below. Editing is only detected in kidney, where six adenosines, occurring in codons 691, 700, 701 and 703, are edited.

We examined the potential A-to-I RNA editing of GLI1 mRNA, which has been demonstrated in humans, by performing RT-PCR on cDNA synthesized from RNA isolated from bowhead retina and kidney tissues. Editing of GLI1 mRNA was identified in the kidney but not in the retina, with degrees of editing at six adenosine positions of between 17% and 24%. Three editing positions occurred within one codon coding for amino acid 701, with the adenosines edited at rates of 10%, 15% and 16%, respectively (Fig 5). The unedited AAA triplet coding for a lysine can be edited to GAA and GAG, encoding a glutamic acid, and GGA and GGG, encoding a glycine residue. In addition, a recoding editing was observed at amino acid position 691, where a serine (AGC) was changed to a glycine (GGC), and at position 700, where threonine (ACC) was substituted by an alanine (GCC) (Fig 5). Finally, editing was also seen at amino acid position 703 (CTA), where the encoded leucine was unaffected. The editing at the second adenosine in the 701 codon is conserved between bowhead and human [95], whereas the remaining five editing sites found in the bowhead GLI1 are novel. We also analysed for A-to-I editing in porcine transcript form the frontal cortex, cerebellum, kidney, liver and muscle. Surprisingly, we did not observe A-to-I editing in the positions where it had been found in human and bowhead.

The glioma-associated oncogene 1 (GLI1) is a transcription factor and a key molecule in the hedgehog signalling pathway, which plays important roles in tumorigenesis and embryonal patterning. Deregulation of the hedgehog signalling leads to developmental defects and different types of tumours. GLI1 is an oncogene, regulation tumour growth, and overexpression of GLI1 leads to cancer [96–98]. Human GLI1 pre-mRNA is subject to A-to-I editing in a number of tissues, and editing is reduced in tumours [96]. The A-to-I editing of the GLI1 transcript leads to an R-to-G amino acid substitution at position 701 [96]. Although the GLI1 coding nucleotide sequence is highly conserved among mammals, the A-to-I editing of it is not necessarily conserved. For example, no GLI1 editing was seen in mice [99]. The editing of human GLI1 pre-mRNA is found in the coding region, more specifically in the sequence encoding the carboxy-terminal half of the protein. The arginine-to-glycine change may affect the structure of the GLI1 protein and the editing of GLI1 has functional consequences. The GLI1 transcript is highly edited in the normal cerebellum, whereas the editing is significantly reduced in cell lines from tumour and basal cell carcinoma samples [96]. The edited GLI1, GLI1-701G, has increased transcriptional activity and also influences GLI1-dependent cellular proliferation [96]. Hence, A-to-I editing of GLI1 contributes to the regulation of the hedgehog signalling pathway. Hyper-editing has been demonstrated in colorectal cancer tissue compared with normal tissues [100].

### SON editing in pigs and bowheads

**Fig 6:**
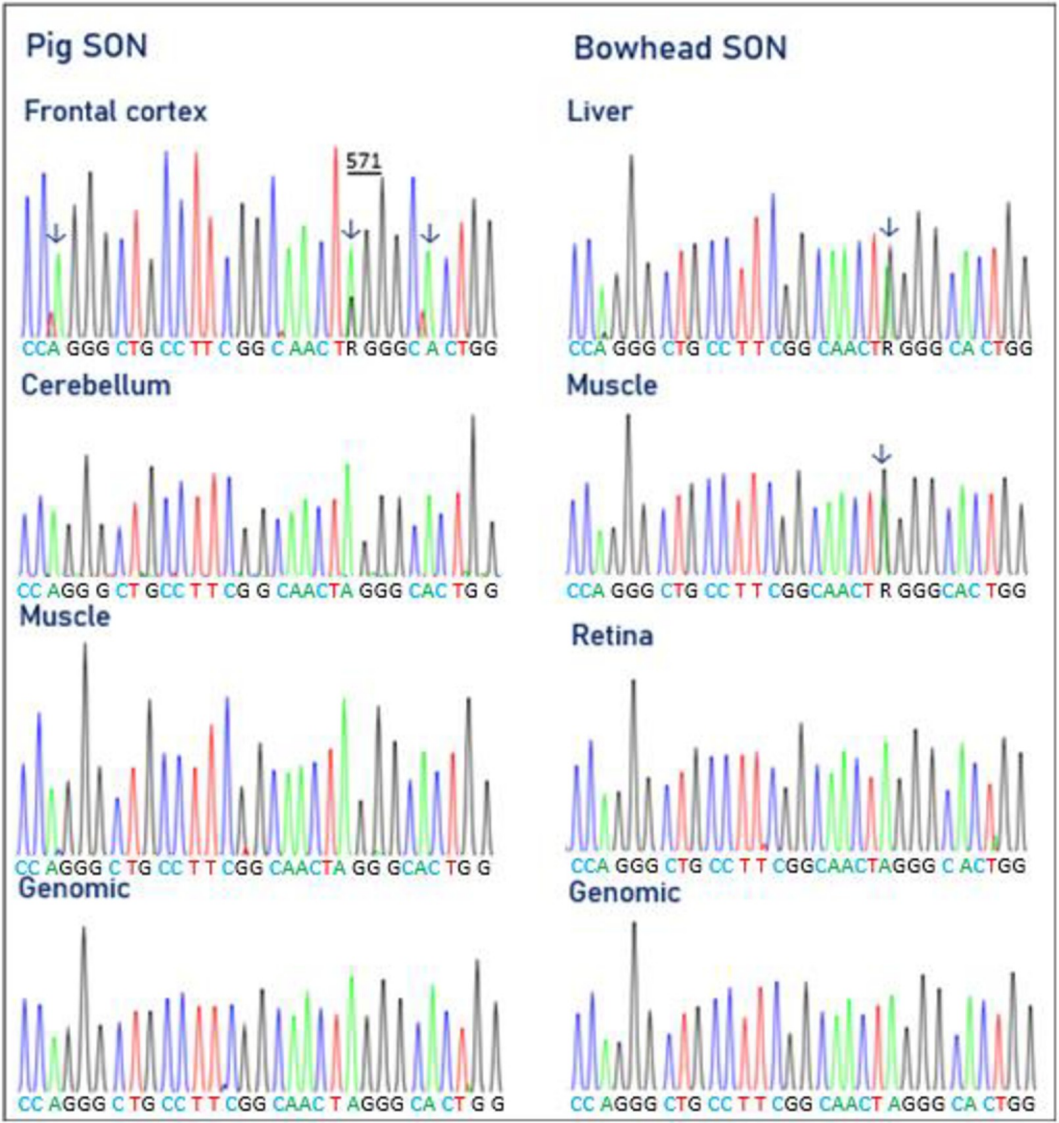
Electropherograms showing SON pre-mRNA editing in pig (left panel) and bowhead (right panel). In pig, SON editing is seen only in frontal cortex at position 571. In bowhead, SON editing is detected in liver and muscle. The adenosines subject to editing are indicated by arrows.

Four A-to-I editing sites were identified in the coding sequence of the human SON pre-mRNA [101]. Editing of one of the four sites results in a recoding of amino acid 571, where an arginine is substituted for by a glycine residue (R571G). This editing site is conserved in mice [59]. The remaining three editing events do not change the encoded amino acids. All four sites in SON pre-mRNA can be edited by ADAR, and two sites in SON (chr 21 + 34923275, new; chr 21 + 34923319, known) can be edited by ADARB1 as well [100]. We evaluated the potential editing of SON mRNA in the pig and bowhead whale. In SON mRNA from the porcine frontal cortex, we found one conserved editing site with an editing degree of approximately 25% (Fig 6). No editing was observed in cerebellum or muscle. Surprisingly, we found editing of the same site conserved in bowhead SON mRNA from muscle and liver but not in the retina (Fig 6 and Table 1). Bakhtiarizadeh et al. [61] recently demonstrated conservation of SON editing in cows. SON functions as an essential DNA-binding protein localized to nuclear speckles and is involved in pre-mRNA splicing [102, 103]. The A-to-I (R571G) editing may modulate the function of SON.

### HTR2C editing in bowheads

**Fig 7:**
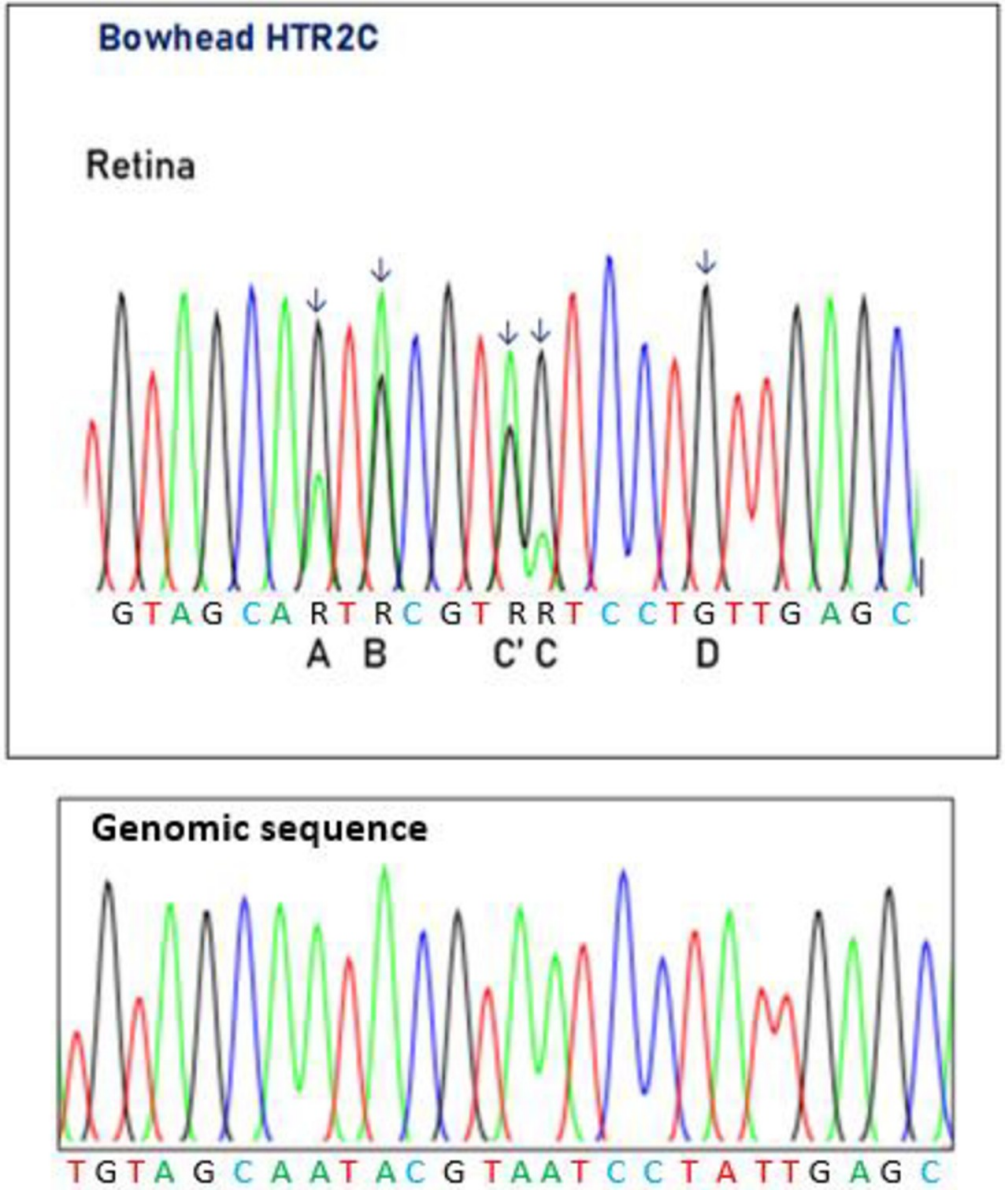
Electropherogram showing HTR2C pre-mRNA in bowhead retina. The adenosines in positions A, B, C’, C and D are all subject to A-to-I editing (arrows). A very high editing degree of 100% is seen for position D.

We examined the A-to-I RNA editing of HTR2C mRNA, which encodes the 5’-hydroxytryptamin (serotonin) receptor 2C, a G protein-coupled protein by performing RT-PCR on cDNA synthesized from the bowhead retina and kidney. Such editing has been demonstrated in humans. Editing of the bowhead HTR2C mRNA was conserved (Table 1) and was identified only in the retina, with editing degrees at five adenosine positions of between 40% and 100%. The editing degrees in the five adenosine positions A, B, C’, C and D were 70%, 42%, 41%, 81% and 100%, respectively (Fig 7). Larsen et al. [33] previously described conservation of A-to-I editing of the porcine HTR2C mRNA. The editing values identified in all five adenosine positions in the HTR2C mRNA were similar to those found in most porcine brain tissues [33]. A-to-I editing is most abundant in the central nervous system. Here, we also report on editing of HTR2C in the bowhead whale retina. The editing of HTR2C can potentially result in 32 different HTR2C mRNAs encoding 24 different protein isoforms. At present, however, we do not have any information about the number of HTR2C isoforms in the bowhead.

### Porcine HTT 3’UTR editing

Using RNA-seq, we identified five A-to-I editing sites in the 3’UTR of the porcine HTT mRNA encoding the huntingtin protein. The editing was confirmed by PCR amplification of the sequences harbouring the edited adenosines. The adenosines subject to editing are located at positions 9649, 9674, 10711, 11796 and HTT-11809 in the GenBank Accession No. NM_213964 (S6 Fig). The adenosines at positions 9649 and 9674 are situated close to the stop codon of the coding sequences of HTT, whereas the other three are distributed further downstream in the 3’UTR. The editing degrees were examined in six different pig tissues and editing was found in cerebellum, frontal cortex and liver at different rates in the five editing sites (S7 and S8 Figs). The editing rates were ranging from 0% to 100% (S2 Table). None of the editing sites have been found in the human HTT 3’UTR. We searched for microRNA binding sites known from the human HTT 3’UTR sequence and found that the binding sequence for miR-216b is completely conserved between human and pig. However, none of the five editing sites identified within the porcine HTT 3’UTR are located in a microRNA recognition sequence. Alignment of the human and the porcine HTT 3’UTR reveals only partial homology with the entire sequence. In particular, the 3’end of the 3’UTR shows a very high homology, and some of the polyadenylation signal sequences (AATGAA, AATGAA and ATAAA) previously reported in the human and mouse HTT 3’UTR are conserved with the porcine sequence [104].

### ADAR2 editing and GRIA2 editing inducer element (EIE) conservation

ADAR2 pre-mRNA is subjected to self-editing, where ADAR2 catalyzes the editing of itself. This self-editing, described in rats and humans, generates alternative splicing variants [105]. The alternative splicing of ADAR2 can occur at nine splicing sites on ADAR2 pre-mRNA. From these nine sites, numerous alternative splicing variants with various catalytic activities are generated. An *in silico* analysis revealed conservation of five potential A-to-I editing sites in the ADAR2 mRNA between the four species compared: pigs, bowhead whales, humans and rats (S9A Fig). A very high nucleotide homology of this ADAR2 sequence is also found between pig and human (S9B Fig). The conserved sequence containing these five sites is located at the 3’-terminal end of intron 3 in the ADAR2 mRNA. We examined the editing pattern of ADAR2 mRNA in pigs and bowheads. In bowheads, we found that only one of the five adenosines edited in humans and rats, namely the adenosine at position –1, was edited: in the bowhead liver (20%) and muscle (21%) but not in the kidney (S10 Fig). Our analysis did not include RNA from bowhead brain tissues, so we cannot exclude the possibility of editing of all five adenosines in the bowhead ADAR2 mRNA. In the pig, four adenosines, at positions –2, -1, + 10 and +24, were edited in frontal cortex and cerebellum, while the adenosine at position +23 was unedited in the examined tissues and organs (S10 Fig). The estimated editing degrees in ADAR2 mRNA from the frontal cortex were as follows: -2 (23%), -1 (55%), +10 (73%) and +24 (75%). In the cerebellum, the values were -2 (16%), -1 (54%), +10 (60%) and +24 (30%). Interestingly, in liver and muscle we only observed editing in position -1 (5–10%). It is likely that the greater number of edited sites among ADAR2 mRNA sites in porcine brain tissues compared to liver tissue might reflect a greater variety of splicing of ADAR2 in brain tissues.

Fu et al. [106] described that RNA editing of ADAR2 leading to shifts in the relative abundance of active and inactive splicing variants of ADAR2 might reduce the ADAR2 editing activity in glioma. An increased expression of ADAR2 splicing variants with low enzyme activity causes reduced RNA editing of the GluA2 subunit at the glutamine/arginine site in glioma.

Using *in silico* analyses, we identified a sequence in the porcine GRIA2 gene highly homologous to a previously described intronic editing inducer element (EIE) [107]. The porcine sequence of 54 nucleotides, shown in Fig 7B, is located in intron 11, 21 nucleotides downstream of the GT intron splice site. The nucleotide sequence shows almost complete conservation with an identity with the human sequence of 96%. The EIE forms a long double-stranded RNA prone to hyper-editing. The long stem formed by the EIE very likely stabilizes the shorter stem formed by the exon 11 and intron 11 sequence [107]. The effects of editing at the GRIA2 Q/R site are dependent on this conserved 45-bp EIE [107].

### ADAR expression in bowheads and pigs

Using in silico analysis in the bowhead sequence database (http://www.bowhead-whale.org/), we identified an ADAR sequence (bmy_09709). A subsequent blastx analysis revealed that the identified ADAR sequence is most similar to ADAR1. The relative expression of bowhead ADAR1 was determined by real-time quantitative PCR in various organs and tissues from bowheads. The highest expression was seen in the retina, optical nerve and kidney, whereas moderate expression was seen in the liver, and low expression in muscle tissue and in the lens (S11A Fig).

The spatial expression of porcine ADAR2 was determined by RNA-Seq as previously described Farajzadeh et al. [108]. The ADAR sequence identified was most similar to ADAR2. Our results show that ADAR2 mRNA expression was seen in the brain tissues (hypothalamus, cerebellum, frontal cortex and occipital cortex), spleen and lung, whereas moderate expression was observed in liver and kidney tissues and heart and skeletal muscle (S11B Fig). In cows, the expression of ADAR mRNA was high in the brain, lung and spleen and lowest in heart and skeletal muscle, which is in accordance with Bakhtiarizadeh et al. [61] reporting that these tissues had the lowest numbers of editing sites. We also examined ADAR2 expression during aging in pigs. Four different tissues from Landrace pigs with the ages 1 year (3 individuals), 7 years (1 individual), 11 years (1 individual) and 12 years (1 individual) were included in the expression analysis. GAPDH was used as a reference gene. Similar levels of ADAR transcript were found in the cerebellum, frontal cortex, spinal cord and kidney (S12A–S12D Fig). In the cerebellum and frontal cortex, no significant changes in ADAR mRNA expression were observed during aging (S12A and S12B Fig). There was a tendency to an increase in ADAR mRNA expression with age in the kidney and conversely a tendency to reduced ADAR expression in the spinal cord with aging (S12C Fig). Clearly, more individuals of older ages are needed, but they are difficult to find. Recently, Zaidan et al. [109] reported age-dependent A-to-I editing in rat brain and that editing is region-specific, and also sensitive to stress.

## Conclusion

A-to-I RNA editing is an evolutionary conserved post-transcriptional mechanism mediated by ADAR enzymes. The A-to-I editing of pre-mRNA leads to increased diversification of the transcriptome and the proteome. Many editing sites have recently been discovered in humans and other species, but the editing rate of particular sites and how the editing is regulated is not completely understood.

To our knowledge, our study is the second to describe A-to-I RNA editing in whales. Editing has previously been reported for minke whale [110]. By RT-PCR and comparison to RNA-Seq and DNA-Seq data from the Bowhead whale genome resource [45], we identified several conserved recoding sites in mRNAs that encode NEIL1, COG3, GRIA2, FLNA, FLNB, IGFBP7, AZIN1, BLCAP, GLI1, SON and HTR2C. All of the editing sites were subsequently identified by comparing mRNA and genomic sequences from the same bowhead individual. We have also genotyped the implicated genome sequences for other individuals. In this way, we have ensured that the identified adenosine to inosine changes are arising from RNA editing and not from SNPs. However, not all adenosine positions edited in the human pre-mRNAs are conserved in the bowhead. This is exemplified by NEIL1 pre-mRNA, with two edited adenosines in the human mRNA but only one adenosine subject to editing in the whale counterpart. Similarly, only one out of four editing sites in human SON mRNA can be found in the bowhead and porcine counterparts. For some of the mRNAs, we observed tissue-specific editing, such as for AZIN1, where editing is found only in the liver. For the bowhead HTR2C pre-mRNA, all five adenosines edited in humans are conserved, and the levels of editing for the individual adenosines seem to be similar to those found for the porcine HTR2C pre-mRNA. The amino acid changes resulting from editing found in the porcine NEIL1, COG3, GRIA2, FLNA, FLNB, IGFBP7, AZIN1, BLCAP, GLI1, SON and HTR2C are conserved in whales, pigs and humans. This suggests that evolutionarily conserved RNA editing sites may be important for gene and protein function of an individual. The demonstrated A-to-I editing is potentially contributing to increased proteome variation. We have performed mass spectrometry analysis of bowhead eye and liver. We have searched the two proteomes for peptide isoforms arising from recoding of NEIL1, COG3, GRIA2, FLNA, FLNB, IGFBP7, AZIN1, BLCAP, GLI1, SON and HTR2C mRNAs. However, we did not identify any peptides translated from recoded mRNA. This might be due to absence or low presence of expression of the individual proteins in the analyzed tissues. Therefore, we do not have any evidence for proteomic changes of the edited bowhead mRNAs. Another consequence of A-to-I mRNA editing is nuclear retention, which is promoted by this modification [111–113]. Hence, retention of some A-to-I edited pre-mRNAs may lead to lack of proteomic change. Nuclear retention of edited mRNAs can also regulate gene expression [114].

There are also differences in the A-to-I editing of certain pre-mRNAs. This is illustrated by the NEIL1 mRNA from the bowhead, which is edited in only one adenosine position (the second) in the AAA triplet encoding lysine 242, whereas in pigs and humans both the second and the third adenosines are edited. In addition, the editing degree of 100% in bowhead NEIL1 mRNA is very high compared with the degrees of editing found in pig and human NEIL1. The implications of this are not known.

The ADAR1- and ADAR2-induced RNA editing in cancer has recently been reviewed by Gatsiou et al. [115]. Collectively, several cancer-associated transcripts from oncogenes and tumour suppressors display dysregulated A-to-I editing. Of special interest are the pre-mRNAs encoding AZIN1, BLCAP, SON and GLI1, all of which display dysregulation in various cancer types. Comparisons across species–human, pig, bowhead, tissues and transcripts reveals differences in A-to-I editing patterns of certain adenosines. For example, some are absent in the bowhead whale or display different editing rates. Whether this contributes to cancer resistance is not known, but functional studies could help to clarify this. Pre-mRNA editing sites related to the development of cancer that are conserved and missing in the bowhead are interesting subjects for further investigation. In future studies, we will perform mass spectroscopy analyses of bowhead tissues to investigate whether isoforms of the various pre-mRNAs generated by editing are present.

Our study identifies some putative functionally important RNA editing sites in pigs, and further studies are needed to reveal the functional changes in RNA editing event genes and whether they might play roles in economically beneficial traits and diseases in pig. No editing of NEIL1, GRIA2 or KCNA1 (Kv1.1) pre-mRNAs was found in shark sequences.

Voltage-gated potassium (Kv1.1) channels are subject to A-to-I editing, generating a channel with a single amino acid substitution located in the inner pore cavity (Kv1.1 I400V). Kv1.1 channels are regulators of neural activity. The A-to-I editing leads to a loss of function, as reduced Kv1.1 channel expression is found at the cell surface [116]. However, Decher et al. [117] showed that channels containing edited Kv1.1 I400V are resistant to blockage by endogenous lipids and that this edit can therefore be regarded as a gain of function. We did not observe any editing of Kv1.1 in brain tissue from the spiny dogfish and Greenland shark. The reasons for this lack of editing and the physiological consequences of it are not known. A cross-species analysis of RNA editing in several tissues demonstrated that species, rather than tissue type, is the primary determinant of editing levels [5]. This suggests stronger cis-directed regulation of RNA editing for most sites, although the small set of conserved coding sites are under stronger trans-regulation. We searched for cis-regulatory sequences in shark genomes, but we did not succeed in identifying any.

A-to-I RNA editing resulting in recoding contributes to a higher proteomic diversity. This is exemplified by a study demonstrating that A-to-I editing leads to higher proteomic diversity in breast cancer [36]. Editing of the COPA transcript increases proliferation, migration, and invasion of cancer cells.

## Supporting information

S1 Fig A-to-I editing of COG3 mRNA in bowhead muscle, kidney, liver, optical nerve and retina. The adenosine (*) in the ATT codon encoding I635 is subject to A-to-I editing in COG3 mRNA from all tissues analyzed (ATT = Ile to GTT = Val). In addition, the adenosine in the third position in the GAA (634) codon is only edited in liver (+). The unedited GAA codon encodes a Glu residue, as does the edited GAG codon. In addition, we also found editing in codon 621 (GAA) marked by **. This editing is not recoding. R = A/G.
(DOCX)

S2 Fig A-to-I editing of shark GRIA2 mRNA. An asterisk indicates the adenosine subjected to editing. A vertical bar indicates the codon 586. R = A/G.
(DOCX)

S3 Fig A-to-I editing of bowhead FLNB mRNA in muscle, retina, liver and kidney. PCR amplicons were sequenced in both directions, forward and reverse. Electropherograms shown represent partial FLNB sequences. An asterisk below the sequence marks the edited adenosine. R = A/G.
(DOCX)

S4 Fig Electropherograms showing the partial bowhead IGFBP7 mRNA sequence. A-to-I editing in the AGG codon for residue R78 (* left) and the AAG codon for K95 (* left). Vertical bars indicate the codons affected by A-to-I editing. R = A/G.
(DOCX)

S5 Fig Amino acid sequence alignment of human and bowhead antizyme inhibitor 1 (AZIN1). Identical amino acids are indicated by asterisks and difference are shown in green. The recoded amino acid at position 367 in the human AZIN1 sequence is shown in yellow.
(DOCX)

S6 Fig Nucleotide sequence of the porcine HTT 3’UTR derived from GenBank access No. NM_213964. The stop codon TGA is shown in a red box. The edited nucleotides are displayed as blue-boxed underlined letters. Two putative polyadenylation sequences are presented in yellow marked letters.
(DOCX)

S7 Fig Electropherograms showing pig HTT pre-mRNA editing in the 3’UTR region in adenosine positions 9649, 9674 and 10711 (NM_213964) in cerebellum, frontal cortex, liver and lung. Arrows indicate edited adenosine positions. R = A/G; Y = C/T.
(DOCX)

S8 Fig Electropherograms showing pig HTT pre-mRNA editing in the 3’UTR region in adenosine positions 11796 and 11809 (NM_213964) in cerebellum, frontal cortex and liver. Edited adenosine positions are indicated by arrows. R = A/G.
(DOCX)

S9 Fig A) Nucleotide alignment of partial ADAR2 sequences from pig, bowhead, human and rat. Boxed letters indicate five adenosines subject to A-to-I editing. B) Nucleotide alignment of GRIA2 EIE showing a very high homology of this regulatory sequence.
(DOCX)

S10 Fig A-to-I editing of ADAR pre-mRNA in bowhead and pig. R = A/G.
(DOCX)

S11 Fig A) ADAR1 mRNA expression across bowhead tissues determined by real-time quantitative PCR. Results are from one individual. B) Expression of porcine ADAR mRNA determined by RNA-Seq. Relative abundance of ADAR is reported in FPKM (Fragments Per Kilobase Million). The tissues presented are occipital cortex (OCC), frontal cortex (FCO), cerebellum (CBE), hypothalamus (HYP), heart (HEA), lung (LUN), musculus longissimus dorsii (LDO), liver (LIV), kidney (KID) and spleen (SPL).
(DOCX)

S12 Fig Relative expression of porcine ADAR mRNA in cerebellum, CBE (B), frontal cortex, FCO (C), spinal cord, SPC (D), and kidney, KID (E). Expression was determined in three individuals of 1 year of age, one individual of 7 years, one individual of 11 years, and one individual of 12 years. GAPDH was used as a reference gene.
(DOCX)

S1 Table Primers used in the analyses of selected edited mRNAs from pig (*Sus scrofa*) and bowhead (*Balaena mysticetus*). (DOCX)

S2 Table Percent editing in porcine HTT 3’UTR adenosine positions. (DOCX)

## Abbreviations

ADAR: adenosine deaminase acting on double-stranded RNA
ADAR2: adenosine deaminase acting on double-stranded RNA 2
ALS: amyotrophic lateral sclerosis
AZIN1: antizyme inhibitor 1
BLCAP: bladder cancer-associated protein
COG3: component of oligomeric Golgi complex 3
ER: endoplasmic reticulum
FLNA: filamin A
FLNB: filamin B
GLI1: glioma-associated oncogene 1
GRIA2: glutamate ionotropic receptor AMPA type subunit 2
HTR2C: 5-hydroxytryptamine receptor 2C
IGFBP7: Insulin Like Growth Factor Binding Protein 7
NEIL1: Nei-like DNA glycosylase 1
SINE: short interspersed nuclear element
SNP: single nucleotide polymorphism
SON: SON DNA binding protein
