# Supplementary material for: Conservation of A-to-I RNA editing in bowhead whale and pig
Source: PLoS One. 2021 Dec 9;16(12):e0260081. doi: 10.1371/journal.pone.0260081 (PMC8659423; doi:10.1371/journal.pone.0260081)
Supplement: S1 Fig — The adenosine (*) in the ATT codon encoding I635 is subject to A-to-I editing in COG3 mRNA from all tissues analyzed (ATT = Ile to GTT = Val). In addition, the adenosine in the third position in the GAA (634) codon is only edited in liver (+). The unedited GAA codon encodes a Glu residue, as does the edited GAG codon. In addition, we also found editing in codon 621 (GAA) marked by **. This editing is not recoding. R = A/G. (DOCX) [file pone.0260081.s001.docx]

|  |
| --- |

**COG3 – Muscle**


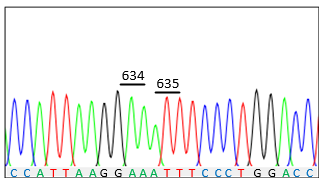


*

Editing at pos. 1 in codon 635 = 0%

**COG3 - Kidney**


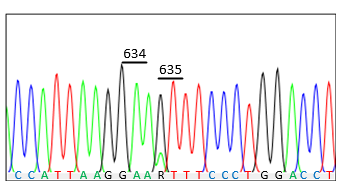


*


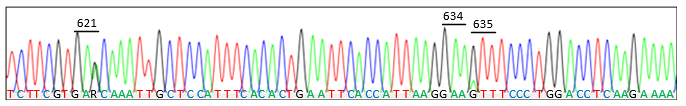


** *

Editing at pos. 1 in codon 635 (*): G/A+G = 20/20 + 5 * 100 = 80 %; editing at pos. 3 in codon 621 (**): 50 %


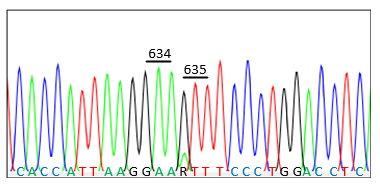


*

Editing at pos. 1 in codon 635: G/A+G = 21/21 + 5 * 100 = 80 %

**Average 80 %**

**COG3 – Liver**


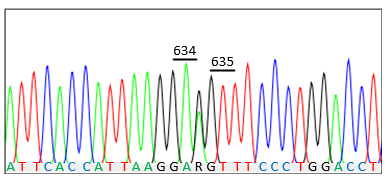


+ *

Editing at pos. 1 in codon 635: **100 %**

Editing at pos. 3 in codon 634: 19/19+13 * 100 = 59 %

**COG3 – Liver**


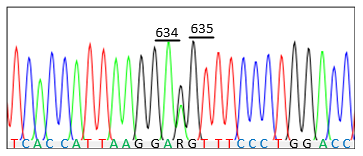


+ *


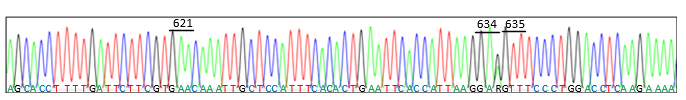


** + *

Editing at pos. 1 in codon 635: **100 %**

Editing at pos. 3 in codon 634: 14/14+9 * 100 = 60 %; Average 60 %

**COG3 - Optical nerve**


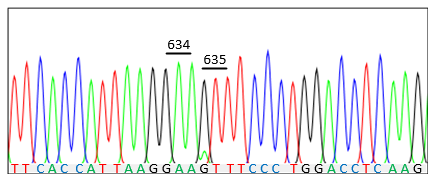


*

Editing at pos. 1 in codon 635: G/A+G = 21/21+3 * 100 = 87.5 %

Editing at pos. 3 in codon 634: 0 %

**COG3 - Optical nerve**


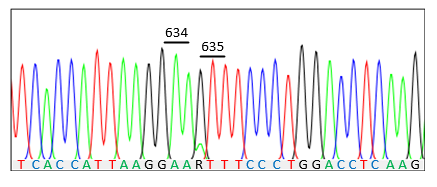


*

Editing at pos. 1 in codon 635: G/A+G = 23/23+4 * 100 = 85.2 %

**Average 86 %**

**COG3 – Retina**


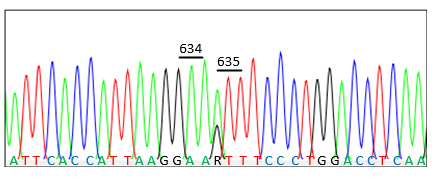


*

Editing at pos. 1 in codon 635: G/A+G = 8/8+16 * 100 = 33 %

**COG3 – Retina**


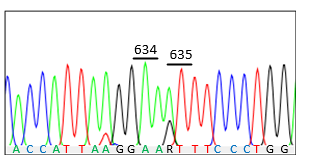


*

Editing at pos. 1: G/A+G = 6/6+15 * 100 = 29 %

**Average 31 %**

pos. 3 in codon 634 pos. 1 in codon 635

Retina 0 % 31 %

Optical nerve 0 % 86 %

Liver 60 % 100 %

Kidney 0 % 80 %

Muscle 0 % 0 %

Editing degree in adenosine position 1 in codon 635 of the bowhead COG3 mRNA

# **Figure S1**
