# Supplementary material for: Conservation of A-to-I RNA editing in bowhead whale and pig
Source: PLoS One. 2021 Dec 9;16(12):e0260081. doi: 10.1371/journal.pone.0260081 (PMC8659423; doi:10.1371/journal.pone.0260081)
Supplement: S2 Fig — An asterisk indicates the adenosine subjected to editing. A vertical bar indicates the codon 586. R = A/G. (DOCX) [file pone.0260081.s002.docx]

| CKA899 | BRAIN-Spiny dogfish-GRIA2 |
| --- | --- |


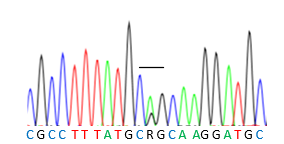


*

| CKA901 | BRAIN-Greenland shark-GRIA2 |
| --- | --- |


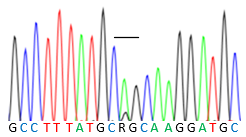


*

| CKA903 | Olfactory bulb-Spiny dogfish-GRIA2 |
| --- | --- |


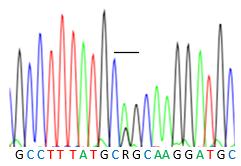


*

| CKA905 | Spinal cord-Greenland shark-GRIA2 |
| --- | --- |


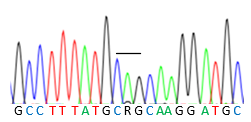


*

| CKA907 | Rosette-Greenland shark-GRIA2 |
| --- | --- |


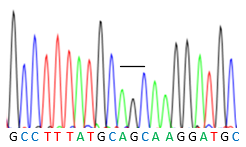


*

# **Figure S2**
