# Supplementary figures and images for: Conservation of A-to-I RNA editing in bowhead whale and pig
Source: PLoS One. 2021 Dec 9;16(12):e0260081. doi: 10.1371/journal.pone.0260081 (PMC8659423; doi:10.1371/journal.pone.0260081)

**Muscle**

Forward


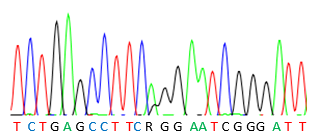


*

Reverse


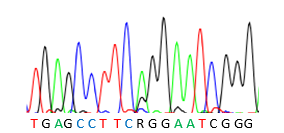


*

**Retina**

Reverse


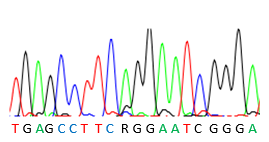


*

**Liver**

Forward


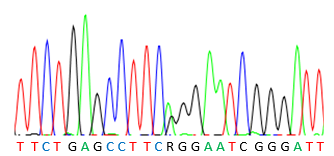


*

Reverse


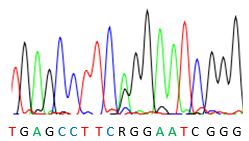


*

**Kidney**

Forward


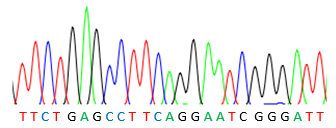


*

Reverse


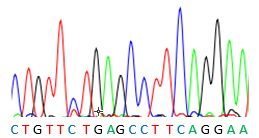


*

**Figure S3**

Supplement: S3 Fig — PCR amplicons were sequenced in both directions, forward and reverse. Electropherograms shown represent partial FLNB sequences. An asterisk below the sequence marks the edited adenosine. R = A/G. (DOCX) [file pone.0260081.s003.docx]

Liver


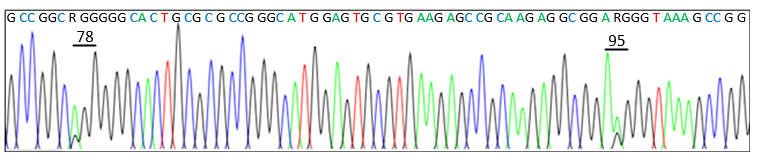


* *

Retina


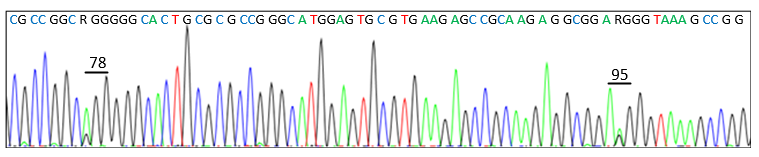


* *

Muscle


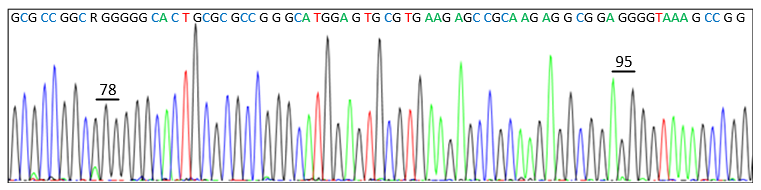


* *

Genomic


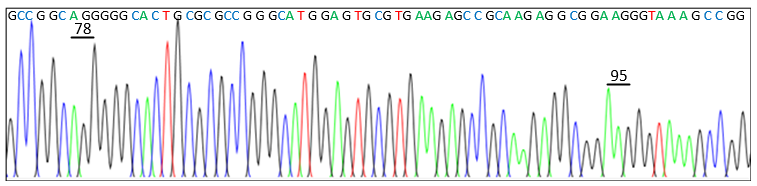


* *

**Figure S4**

Supplement: S4 Fig — A-to-I editing in the AGG codon for residue R78 (* left) and the AAG codon for K95 (* left). Vertical bars indicate the codons affected by A-to-I editing. R = A/G. (DOCX) [file pone.0260081.s004.docx]

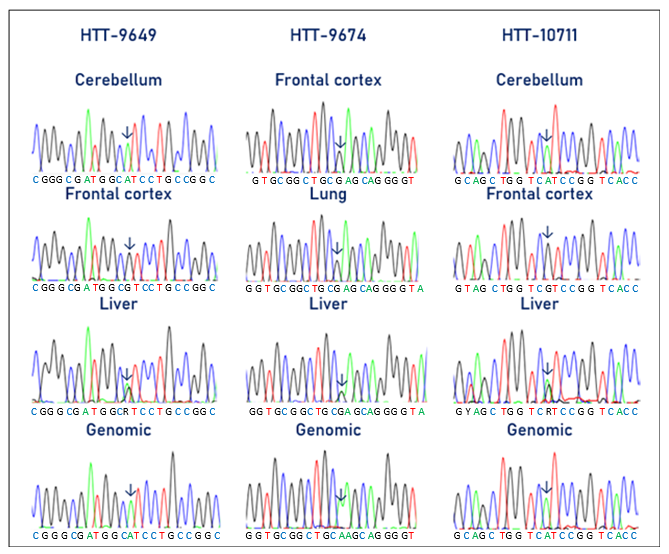


**Figure S7**

Supplement: S7 Fig — Arrows indicate edited adenosine positions. R = A/G; Y = C/T. (DOCX) [file pone.0260081.s007.docx]

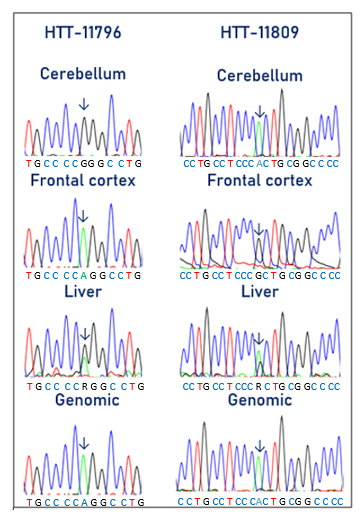


**Figure S8**

Supplement: S8 Fig — Edited adenosine positions are indicated by arrows. R = A/G. (DOCX) [file pone.0260081.s008.docx]

A.


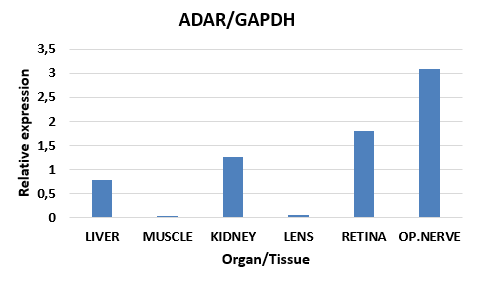


B.

**Figure S11**

Supplement: S11 Fig — A) ADAR1 mRNA expression across bowhead tissues determined by real-time quantitative PCR. Results are from one individual. B) Expression of porcine ADAR mRNA determined by RNA-Seq. Relative abundance of ADAR is reported in FPKM (Fragments Per Kilobase Million). The tissues presented are occipital cortex (OCC), frontal cortex (FCO), cerebellum (CBE), hypothalamus (HYP), heart (HEA), lung (LUN), musculus longissimus dorsii (LDO), liver (LIV), kidney (KID) and spleen (SPL). (DOCX) [file pone.0260081.s011.docx]
