# Supplementary material for: Conservation of A-to-I RNA editing in bowhead whale and pig
Source: PLoS One. 2021 Dec 9;16(12):e0260081. doi: 10.1371/journal.pone.0260081 (PMC8659423; doi:10.1371/journal.pone.0260081)
Supplement: S5 Fig — Identical amino acids are indicated by asterisks and difference are shown in green. The recoded amino acid at position 367 in the human AZIN1 sequence is shown in yellow. (DOCX) [file pone.0260081.s005.docx]

Bowhead AZIN1 MKGFIDDANYSVGLLDEGTNLGNVIDNYVYEHTLTGKNAFFVGDLGKIVKKHSQWQNIVA 60

Human AZIN1 MKGFIDDANYSVGLLDEGTNLGNVIDNYVYEHTLTGKNAFFVGDLGKIVKKHSQWQNVVA 60

********************************************************* **

Bowhead AZIN1 QIKPFYTVKCNSTPAVLEILAALGTGFACSSKTEMALVQELGVSPENIIYISPCKQVSQI 120

Human AZIN1 QIKPFYTVKCNSAPAVLEILAALGTGFACSSKNEMALVQELGVPPENIIYISPCKQVSQI 120

************ ******************* ********** ****************

Bowhead AZIN1 KYAAKVGVNIMTCDNEVELKKIARNHLNAKVLLHIATEDNIGGEEGNMKFGTTLKNCRHL 180

Human AZIN1 KYAAKVGVNILTCDNEIELKKIARNHPNAKVLLHIATEDNIGGEEGNMKFGTTLKNCRHL 180

********** ***** ********* *********************************

Bowhead AZIN1 LECAKELDVQIIGVKFHVSSACKESQVYVHALSDARCVFDMAGEFGFTMNMLDIGGGFTG 240

Human AZIN1 LECAKELDVQIIGVKFHVSSACKESQVYVHALSDARCVFDMAGEIGFTMNMLDIGGGFTG 240

******************************************** ***************

Bowhead AZIN1 TEFQLEEINHVISPLLDVYFPEGSGIKIISEPGSYYVSSAFTLAVNIIAKKVVEN-KFSS 299

Human AZIN1 TEFQLEEVNHVISPLLDIYFPEGSGVKIISEPGSYYVSSAFTLAVNIIAKKVVENDKFPS 300

******* ********* ******* ***************************** ** *

Bowhead AZIN1 GVGKTGSDEPTFMYYMNDGVYGSFASKLSEDLNTIPEVHKKYKEDEPLFTSSLWGPSCDE 359

Human AZIN1 GVEKTGSDEPAFMYYMNDGVYGSFASKLSEDLNTIPEVHKKYKEDEPLFTSSLWGPSCDE 360

** ******* *************************************************

Bowhead AZIN1 LDQIVENCLLPELNVGDWLIFDNMGADSFHEPSAFNDFQRPAIYYMMSYSDWYEMQDAGI 419

Human AZIN1 LDQIVESCLLPELNVGDWLIFDNMGADSFHEPSAFNDFQRPAIYYMMSFSDWYEMQDAGI 420

****** ***************************************** ***********

Bowhead AZIN1 TSDTMMKNFFFVPSCIQLSQEDNFSTEA- 447

Human AZIN1 TSDSMMKNFFFVPSCIQLSQEDSFSAEA- 448

*** ****************** ** **

**Figure S5**
