# Supplementary material for: Conservation of A-to-I RNA editing in bowhead whale and pig
Source: PLoS One. 2021 Dec 9;16(12):e0260081. doi: 10.1371/journal.pone.0260081 (PMC8659423; doi:10.1371/journal.pone.0260081)
Supplement: S6 Fig — The stop codon TGA is shown in a red box. The edited nucleotides are displayed as blue-boxed underlined letters. Two putative polyadenylation sequences are presented in yellow marked letters. (DOCX) [file pone.0260081.s006.docx]

GATGTCCACAGGGTGGCTGCATGCTGAGGCCCACGCGGTGGCAGCGAGGCGGCGGGGACTTTGGGCCTGC

CAGCCCGGTGGCTTGGCATCACGGGCTTCCCTCTGTGGCTTGGGGGCCGGGCGATGGC**G**TCCTGCCGGCA

GCGGTGCGGCTGC**A**AGCAGGGGTACATGCAGGAGGCTCTGGGGTTGAGCCCGAGTCCTCCCAGGGCAGGA

GCAGCTGTGTCGCGAGCCCTCCGTGCCCGAGTCCATTAAATGCCAGTTAGCCAGGTGTTGATCTTCCTCC

TCCTGTCCAGCCTGTTCCTTGGCACCAGGAGTTCTCTACTCTCGCAGGCTGGCCGAGGGCCCCTCTGCTG

TCCCGCAGGCGGGCTTTGGAGCACACCCCTCGCGTCCCCGCGGCGAGGCGAGGGGGCCCGGGGTCTCGTC

TGGGCCGGGCGGGGTGGCTGGGAGCACGACGGCCCGCACCGTTCTTCCCTGTCTGTTTTCTTCTCAGGAT

TTAAAGTTTAATTATATCAGTAAAGAGATTACTTTTAAGGTAACTTTCTATGCCCGTGTACAGTCTGTGC

ATTGCCAAGTCTGCTGCATGCGCGGCCTCGTGTGGCGGGCAGGCCTGTGCCCCTTCCTCTGCCGACAGCT

CCCCCCTGCCCCCTCTCCTCTCATTCACATCTAGTTGGAGTTTAGGGAACGTTGGTGCAGGCCTTCTCCC

CGGGGCACGGACGGCTCTCACAGTGCCCACCCCCCGGGCCCTCTGCTGACCCGGCCACAAGCAGCTGCTG

TCGCTTCCCCGGCGTCCGTCCTGAGCTGGGCCACGGTCTGTCGGGCTGGCTGGTGGGCGTTGGGACCTGC

GTCTCGTGTCTTCTCCGCATGTTGGGGCGCAGAGGTGTCCAGTGGCTTCGAGAGTGTAAGGGCGGTATTT

GTCCCGACCCCTCAGGCAAGACAGAGCTCGGCGTCCTGGGAAAGACCCCAGCCGCTCCTCGGCCCCCTCC

CCAGCTCAGCCCAGACGCGGCGCCTTCTTTCAGCTCAGGGGAGCGTGGGCCTGTGTGTGCGCCCCCAGCC

TCTGCGCGGGGCCAGCCCGGGTTCTGGACCCAGAGGGCCAGATCACCAGATGCAGAGGCGGCCTGGGCTG

CCGGGTGGCCCTGGCCAGGAGCCCCTGGGGCACTTAGGGCGGGCTCTCCATGTCCCCGGCGCAGCTGGTC

**G**TCCGGTCACCCTCGTGTTGGCCAGTTTGGTCAGAGCTCAAGTGCGGTCAGGTTTGGTGTTTCGTCCTAA

GTGGAGGTGATGTGGATGCCGTGTCCCCAGGGCTGCACGGGGTCCTTGGGGCCCATGCGCCACCCTCTGT

CTGTGCGACATCTCGCCACAGCGCACAGCCCGTCCCCAAAGGCCTTGGGGTCAGAGTGTGCGTGGGGCCA

GGGGAGGCGGTGGGGGCCTCCTCAGAGCCGCCTCTGGTTTCCCACCAGCCTCCAGCAGAGGCCTCGCCGG

GCCGGAGGGTGCCCAGTAGAGAGGAGTCTGGCCCGAGCAGGGCCTTGCCGGAGGGACGCCGACTGAACCA

GACGTGAGGAAGTGACCGCTCTCCTGTTCCCTTTGGCCTCCCGCCTCTCACGCTAGCTCTTCCCGTCGGG

CACCTGCTGGGATGTTTGCGAGCGCAGGAAGGTTCTAGAGCTGCTTCCCTTGCCGCCCCCCGCGTTCTTG

GAGCAGCTCCGAGACGGGGGTGCTGCAGGCCGACGTGGTCCCGCTCGGGCTGTCGGCTCGTTAGCGGTAA

CGCTGTGCGTGTACAAGCGGGGGCGCGGCACTGGGCTCGGCTGAGTTGACTCGGCCCCTCGTCGACCGTG

TGACCCGGAGCTGGGCTCCAGCCTCAGGCGCCGACCCGGAGACCCGCCCGTGCGCTGCCCACGTGCGTGC

ATTGGTGAGGGAGGTGGTTTCAGCCGGGTGAGCGTGTGAGACGCAAGGGCTAATTCTGTATCTGCAGACA

CGCCCGGCCGAGGAACGGCCAGGTCCTCGCGGAAGCCGACTTTCCCGAGCCCTGCCACGTGGTCCGCCGC

GGGGCCTGGGGTGCTCGTTAAAACCCCGCTGAGGACTCGCCCACCTTGTCGGCTCCGCAGGAGGGGCTGA

GACGGGATCTGGTTCTTGAAAGCCCCTGGCCGGCACCCTCTCGTTGCCGATTTTGTTGAAGATGTCTTTC

GCTTGGTTGTCAGGTTCGTCTGGGAGGCGGGGTCCTGCTGGGAATCAGGACGTAGGAGTGAAACCAGGGT

GGAGTTGGCCGCGACGCGCCTGCGCAGCCTGCCCC**G**GGCCTGCCTCCC**G**CTGCGGCCCCGCGCTGCCCGG

GCCTCCCAGGTGCCAAGGGCCCTTGGCTGTGCACCCACTTCTGCCGGCTCGAGCTCCCTTCGGAGGAAGG

GGTGTGCTGGACACCCAGATGCTCAGAACGCAGCCCCAGGAGGCCTGCCGCACGCAGACCCACTTCGTGG

CCGGGACGCTGCCAAGCCCTCGGGTATGGGCACCGAGGGGGGCCCTGCTTCGGTTCAGGTGGGACGTCCC

GAAGCGGGGTAGCCCCTTTGGGCTGCACCAGCTGTCGCACATGGTGTGCGCGGCTGGACTCTGCTTCCTC

CCAACCCCGCGAGCATCTTTCCTGCCGGGTCCTCTCTGGGCCTCGTGGCCCTCCCTCCGTGGTCAGTGCC

CGGCCAGCGGGAGGCCTGTCTGCTGGCGCGATGGGCAGGGAGCTGCAGAGCTGGGGGTGTGCGCGAGAGG

GGTCAGGCTCAGCAGGGAGGAGCCCCGCTCCAACGGTGGTGACCACCAAAGGTGGTCCGGAAGGGCCAAG

GACACTCTGCCCACCTGCTGTCCCTCAGCGTCACCCCACTGGGGGCGTCTGGTCCTGGACGGTCACCACA

GTGTTTGTCATTGTCAGTGGGTGACCTCTGGCTTTGGAGGGTTGTAGCCGAAGGTGGTTTGGCTCTGAGC

TAAGTGGGGCCACTGCCCGTGCCTGCTGCCTCGCGCATGGCAGAGTGGCTTTCGGCAGGGTTTGAACTCG

TACTGTGCCATTGCTGTCCCCCACCCCTACCCCCGGTCGCCGCCTCGCTGGGAGCCGGCAGGGCTGTGAA

GGGCACGTCCCGGAGCCTGGCCCAGACCTGTTTCTGAGAGGAGAAAGAGGGTCTGACGTGAGATGTCTAT

TTTAATTTTTTAACTGCTGCAAACATTGTACATCTGAAT

**Figure S6**
