# Supplementary material for: Conservation of A-to-I RNA editing in bowhead whale and pig
Source: PLoS One. 2021 Dec 9;16(12):e0260081. doi: 10.1371/journal.pone.0260081 (PMC8659423; doi:10.1371/journal.pone.0260081)
Supplement: S9 Fig — A) Nucleotide alignment of partial ADAR2 sequences from pig, bowhead, human and rat. Boxed letters indicate five adenosines subject to A-to-I editing. B) Nucleotide alignment of GRIA2 EIE showing a very high homology of this regulatory sequence. (DOCX) [file pone.0260081.s009.docx]

**Figure S9**
