# Supplementary material for: Conservation of A-to-I RNA editing in bowhead whale and pig
Source: PLoS One. 2021 Dec 9;16(12):e0260081. doi: 10.1371/journal.pone.0260081 (PMC8659423; doi:10.1371/journal.pone.0260081)
Supplement: S10 Fig — R = A/G. (DOCX) [file pone.0260081.s010.docx]

Bowhead Liver


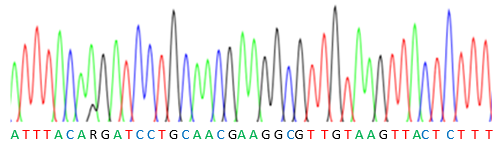


-1

Bowhead Kidney


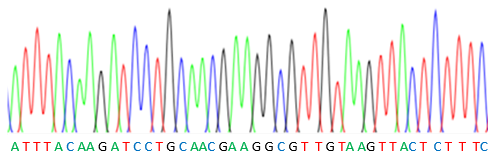


-1

Bowhead Muscle


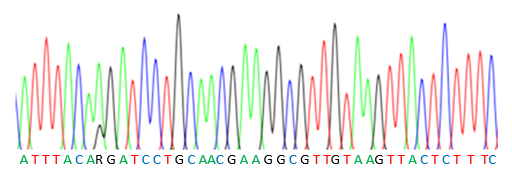


-1

Bowhead Genomic


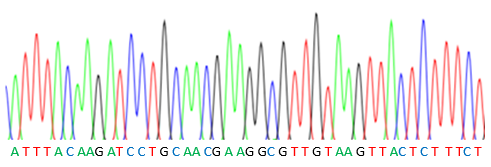


-1

Pig Frontal cortex


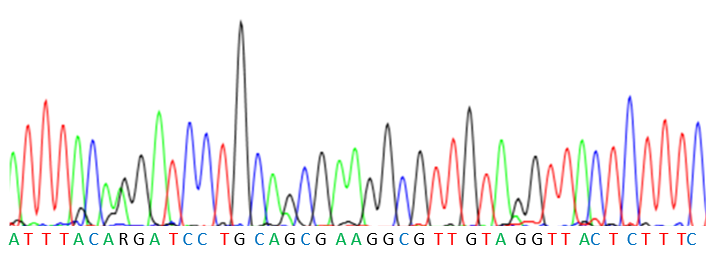


-2 -1 +10 +23+24

Pig Cerebellum


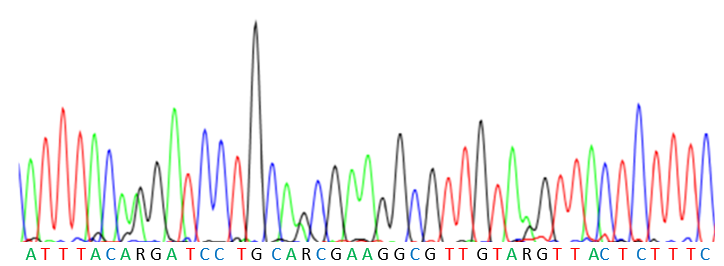


-2 -1 +10 +23+24

Pig Muscle


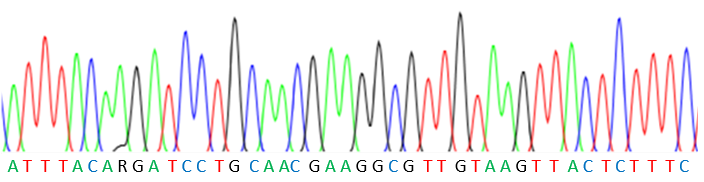


-2 -1 +10 +23+24

Pig Liver


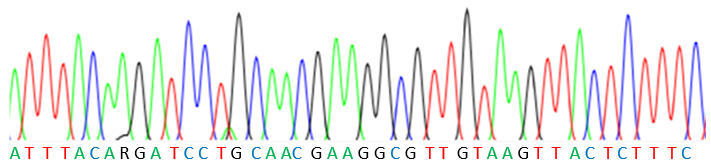


-2 -1 +10 +23+24

Pig Genomic


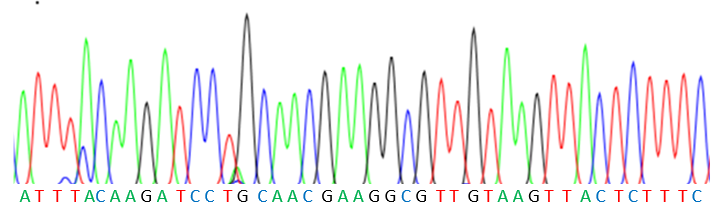


-2 -1 +10 +23+24

**Conclusion:**

In bowhead, only one adenosine is edited at position -1; in liver (20 %) and muscle (21 %).

In pig, four adenosines are edited at positions: -2, -1, +10 and +24.

FCO: positions: -2 (23 %), -1 (55 %), +10 (73 %) and +24 (75 %).

CBE: positions: -2 (16 %), -1 (54 %), +10 (60 %) and +24 (30 %).

LIV: only editing in position -1 (10 %)

MUSC: only editing in position -1 (5 %)

**Figure S10**
