# Supplementary material for: Conservation of A-to-I RNA editing in bowhead whale and pig
Source: PLoS One. 2021 Dec 9;16(12):e0260081. doi: 10.1371/journal.pone.0260081 (PMC8659423; doi:10.1371/journal.pone.0260081)
Supplement: S12 Fig — Relative expression of porcine ADAR mRNA in cerebellum, CBE (B), frontal cortex, FCO (C), spinal cord, SPC (D), and kidney, KID (E). Expression was determined in three individuals of 1 year of age, one individual of 7 years, one individual of 11 years, and one individual of 12 years. GAPDH was used as a reference gene. (DOCX) [file pone.0260081.s012.docx]

A.

B.

C.

D.

**Figure S12**
