# Supplementary material for: Conservation of A-to-I RNA editing in bowhead whale and pig
Source: PLoS One. 2021 Dec 9;16(12):e0260081. doi: 10.1371/journal.pone.0260081 (PMC8659423; doi:10.1371/journal.pone.0260081)
Supplement: S1 Table — (DOCX) [file pone.0260081.s013.docx]

**Table S1**

Primer Sequence (5’-3’) Amplicon size

SSNEIL1-AIF GATCCTACACCGGCTGAGGATACC 318 bp

SSNEIL1-AIR GGTCCAGGATCCCCCTGGAACCAGATG

BMNEIL1-EF1 AGCCCGGAGCTGACCCTGAGC 238 bp

BMNEIL1-ER1 CTGGAACCAGATGGTGCGGCCCTG

SANEIL1-AIF TGAGAAGGCGAGGACTGTGTTGGAA 322 bp

SANEIL1-AIR CTTGGGTTTGGGAGTCTTGGATCCTT

SSCOG3-AIF CACAGGAGGCCTTGTCTGCCTGC 288 bp

SSCOG3-AIR GGAGTACCCTCCAACAAGAACTC

BMCOG3-AIF CAGATGTATAGATAGGGCAGTAT 425 bp

BMCOG3-AIR GCTCTACGAACAGCTTGGTCTGCTGC

SSGRIA2-AIF GCTGACCTTGCGGTTGCTCCA 250 bp

SSGRIA2-AIR CCCTTGCTGCATGAGAGCTCC

SAGRIA2-F GACTTTTCCAAACCTTCATGAGT 245 bp

SAGRIA2-R CAACCTTGGCGAAATATCGCATCC

BMFLNA-AIF GTCTCAGTCAAGTTCAACGAGGAAC 207 bp

BMFLNA-AIR CAGGGCTCCTGAGGGGCTGTGCAC

BMAZIN1-F ATGAAAGGATTTATTGACGATGCA 1032 bp

BMAZIN1-R TTAAGCTTCAGTGGAAAAGTTGTC

BMAZIN1-EDF GGTTCTTTTGCAAGTAAGCTGTCTG 300 bp

BMAZIN1-EDR TGTGTCTGAAGTAATTCCAGCATC

BMBLCAP-EDF TGGTGATCCCGGGCTGACAGC 303 bp

BMBLCAP-EDR GGCACAAATTGTGCAAGGCTTCCG

BMGLI1-EDF CGGTTCAAGAGCTTGGGTTGTGTC 303 bp

BMGLI1-EDR ACCAGGCCCAAGAGGCAGGGA

BMHTR2C-AIF ATGCACCTCTGCGCTATTTCGCTG 245 bp

BMHTR2C-AIR GCGAAGAACGTGGATAGTTACACA

SSSON-AIF GGGCAGCCTGAGGCAGCAATGGTG 276 bp

SSSON-AIR GCACCCCTGTTGCCAAAGGCTGCC

BMKCNB1-AIF GATGACACCAAGTTCAAAAGCATCCC 260 bp

BMKCNB1-AIR CAGAGCCTCTCTCCGCTTGATTGCTT

BMIGFBP7-AIF CTCTCCTCTTCCTCCTCTTCGGA 305 bp

BMIGFBP7-AIR CCGTCGCTGCCGCACACCGGGTA

BMFLNB-AIF AACGGATCATGTGGTAACTACGAG 297 bp

BMFLNB-AIR GCAGAAACATTGCTGGTGTGGGCG

BOWADAR2-F TAGGTGGATAGATAGCTCTGTGG 349 bp

BOWADAR2-R CGGAACTTGGAGTGGCCATTGCTG

PIGADAR2-EDF GGTTAAACGCGAGGCTCCAGACAT 326 bp

PIGADAR2-EDR TGCCCGTTGCTGCCCTCCTCGAGCGG

HTT-F1 CCACAGGGTGGCTGCATGCTG 300 bp

HTT-R1 GTGCCAAGGAACAGGCTGGAC

HTT-F2 TGGGCCTTTGTGTGCGCCCCC 1001 bp

HTT-R2 CGGACCACGTGGCAGGGCTCG

HTT-F3 GGCCGGCACCCTCTCGTTGCC 338 bp

HTT-R3 TTGGCAGCGTCCCGGCCACGA

HTT10711-F CTGCGCGGGGCCAGCCCGGGTTCT 1054 bp

HTT10711-R CCGTCTCAGCCCCTCCTGGGGAGC

BOWADAR-QF GGGCTACGGGAACTGGATA 62 bp

BOWADAR-QR CACTGGGCAGAGGTAAAAATTC

ADAR - PROBE # 63 AGGAGGAG

BMGAPDH-F AACGTGTCCGTCGTGGAT 78 bp

BMGAPDH-R TCAAGAAGGTGGTGAAGCAG

GAPDH- PROBE # 80 CCTGGAGA

PIGADAR-QF TGTGACTACCTGTTTAACGTGTCC 61 bp

PIGADAR-QR GGCCAATGTTTTTAGCCAAG

PIGADAR – PROBE # 82 CTCCTCTG

PIGGAPDH-QF

PIGGAPDH-QR

PIGGAPDH – PROBE # 9

SS = *Sus scrofa* - pig

BM = *Balaena mysticetus* - bowhead

SA = *Squalus acanthius –* spiny dogfish
