# Supplementary material for: Conservation of A-to-I RNA editing in bowhead whale and pig
Source: PLoS One. 2021 Dec 9;16(12):e0260081. doi: 10.1371/journal.pone.0260081 (PMC8659423; doi:10.1371/journal.pone.0260081)
Supplement: S2 Table — (DOCX) [file pone.0260081.s014.docx]

| **Table S2.** | | | | | |  |
| --- | --- | --- | --- | --- | --- | --- |
|  | CBE | FCO | OCC | LIV | LUN | MUS |
| HTT-9649 | 0 | 100 | 100 | 40 | 0 | 0 |
| HTT-9674 | 100 | 0 | 0 | 50 | 100 | 100 |
| HTT-10711 | 0 | 100 | 100 | 40 | 0 | 0 |
| HTT-11796 | 100 | 0 | 40 | 60 | 100 | 100 |
| HTT-11809 | 0 | 100 | 100 | 35 | 0 | 0 |
|  |  |  |  |  |  |  |
|  |  |  |  |  |  |  |
|  | CBE | FCO | OCC | LIV | LUN | MUS |
| HTT-9649 | 0 | 100 | 100 | 40 | 0 | 0 |
| HTT-9674 | 100 | 0 | 0 | 50 | 100 | 100 |
| HTT-10711 | 0 | 100 | 100 | 40 | 0 | 0 |
| HTT-11796 | 100 | 0 | 40 | 60 | 100 | 100 |
| HTT-11809 | 0 | 100 | 100 | 35 | 0 | 0 |
